# Exploration of the pearl millet phospholipase gene family to identify potential candidates for grain quality traits

**DOI:** 10.1186/s12864-024-10504-x

**Published:** 2024-06-10

**Authors:** Mazahar Moin, Pradeep Reddy Bommineni, Wricha Tyagi

**Affiliations:** Cell and Molecular Biology and Trait Engineering, International Crops Research Institute for the Semi-Arid Tropics (ICRISAT), Hyderabad, Patancheru, Telangana 502324 India

**Keywords:** Pearl millet, Phospholipases, PLD, Grain quality, Rancidity

## Abstract

**Background:**

Phospholipases constitute a diverse category of enzymes responsible for the breakdown of phospholipids. Their involvement in signal transduction with a pivotal role in plant development and stress responses is well documented.

**Results:**

In the present investigation, a thorough genome-wide analysis revealed that the pearl millet genome contains at least 44 phospholipase genes distributed across its 7 chromosomes, with chromosome one harbouring the highest number of these genes. The synteny analysis suggested a close genetic relationship of pearl millet phospholipases with that of foxtail millet and sorghum. All identified genes were examined to unravel their gene structures, protein attributes, *cis*-regulatory elements, and expression patterns in two pearl millet genotypes contrasting for rancidity. All the phospholipases have a high alpha-helix content and distorted regions within the predicted secondary structures. Moreover, many of these enzymes possess binding sites for both metal and non-metal ligands. Additionally, the putative promoter regions associated with these genes exhibit multiple copies of *cis*-elements specifically responsive to biotic and abiotic stress factors and signaling molecules. The transcriptional profiling of 44 phospholipase genes in two genotypes contrasting for rancidity across six key tissues during pearl millet growth revealed a predominant expression in grains, followed by seed coat and endosperm. Specifically, the genes *PgPLD-alpha1-1*, *PgPLD-alpha1-5*, *PgPLD-delta1-7a*, *PgPLA1-II-1a*, and *PgPLD-delta1-2a* exhibited notable expression in grains of both the genotypes while showing negligible expression in the other five tissues. The sequence alignment of putative promoters revealed several variations including SNPs and InDels. These variations resulted in modifications to the corresponding *cis*-acting elements, forming distinct transcription factor binding sites suggesting the transcriptional-level regulation for these five genes in pearl millet.

**Conclusions:**

The current study utilized a genome-wide computational analysis to characterize the phospholipase gene family in pearl millet. A comprehensive expression profile of 44 phospholipases led to the identification of five grain-specific candidates. This underscores a potential role for at least these five genes in grain quality traits including the regulation of rancidity in pearl millet. Therefore, this study marks the first exploration highlighting the possible impact of phospholipases towards enhancing agronomic traits in pearl millet.

**Supplementary Information:**

The online version contains supplementary material available at 10.1186/s12864-024-10504-x.

## Background

Pearl millet, an annual crop known for its climatic resilience, thrives on millions of acres worldwide [1]. Its extensive root system enables it to endure in diverse ecological conditions even under limited water conditions [2]. This crop exhibits high photosynthetic efficiency, leading to exceptional productivity and growth in low-nutrient soils, reducing the reliance on chemical fertilizers [2, 3]. Identified as a C4 plant, pearl millet plays a significant role in sequestering global terrestrial carbon, contributing approximately 30% of the total amount, alongside maize and sorghum [4]. The primary constraints on the agricultural productivity of any crop include drought and heat among various environmental challenges. In this context, pearl millet stands out as a resilient crop capable of withstanding relatively high temperatures and reduced precipitation [5, 6]. Hence, it holds promise in addressing the increasing global food demands driven by population growth under adverse environmental conditions. Consequently, it can also serve as an alternative crop within cereal systems amidst changing climatic patterns [7].

Several studies have underscored that the suboptimal yields of pearl millet in certain tropical areas may be attributed to conventional farming methods, particularly inadequate planting densities [8]. Furthermore, disparities in the productivity of crops are linked to factors such as the density of panicles per square meter, the number of grains per panicle, and the weight of individual grains [9, 10]. The most substantial reductions in grain yields are also observed during periods of environmental stress coinciding with flowering and grain-filling stages [11]. This decline in yield is linked to a decrease in both seed quantity per plant and grain weight which is further accredited to a shortened duration of grain filling [12, 13]. All these findings have been instrumental in directing efforts towards improving pearl millet yield. The genetic enhancement of pearl millet to increase its productivity was first accomplished through the integration of hybrid technology and traditional breeding methods for selection [14–16]. In addition to conventional methodologies, it is also essential to pinpoint potential candidate genes that could contribute to enhancing grain-related traits. However, due to its recalcitrance to tissue culture media, progress in genetic improvement involving biotechnological applications utilizing genetic and genome-engineering approaches remains limited.

Despite its remarkable nutritional composition, gluten-free nature and advantageous agronomic traits, pearl millet has not attained widespread popularity on a global scale in comparison to other cereal crops. This can be ascribed to the short shelf life and rapid development of off-flavours in pearl millet flour [17]. The accrual of lipids and the enzymatic activity of phospholipases, lipoxygenases, peroxidases, and polyphenol oxidases contribute to the swift onset of rancidity and undesirable odours in the pearl millet flour [17, 18]. External factors such as oxygen, light exposure, and storage temperatures also impact the longevity of the flour [19, 20]. This oxidative rancidity is further exacerbated by fostering the production of hydroperoxides and secondary metabolites that are accountable for the bitter and musty odour associated with pearl millet flour [21].

Plant phospholipases are commonly categorized into three distinct families, namely PLA, PLC, and PLD. This classification is based on their substrate specificities and the requirement for either metal or non-metal cofactors [22]. PLAs are responsible for breaking down acyl groups, PLCs target the phosphodiester bonds of phospholipids, and PLDs specialize in cleaving terminal phosphodiester bonds [23–25]. PLAs act upon substrates like triacylglycerol (TAG), diacylglycerols (mono- and di-galactosyl diacylglycerols), phosphatidylethanolamine (PE), and phosphatidylcholine (PC). Plant PLCs demonstrate activity on a wide range of substrates [26, 27]. PLDs are recognized for their diverse enzymatic, regulatory, and structural characteristics. Their substrates include PE, PC, phosphatidylserine (PS), phosphatidylinositol (PI), phosphatidylglycerol (PG), and N-acyl phosphatidylethanolamine [24, 28]. PLD serves various regulatory functions in a wide range of plant processes, such as abscisic acid (ABA) signaling, root and seedling growth, root hair patterning, programmed cell death, freezing tolerance, and responses to stress [29–32].

Considerable progress has been made in understanding the regulation of phospholipases. Nevertheless, further exploration of the functional properties of these enzymes and their resultant messengers will contribute to the advancement of our knowledge concerning signaling networks implicated in plant growth, development and productivity, as well as the ability to respond to stress stimuli. Phospholipases also hold a pivotal position in the pathway of rancidity, making them a critical determinant of the extent of rancidity in flour [17]. To prolong the shelf life of the flour, it is imperative to initially evaluate the impact of potential phospholipases on hydrolytic rancidity, followed by managing factors contributing to oxidative rancidity. In line with this objective, the current investigation centres on the comprehensive exploration of the phospholipase gene family, their chromosomal distribution, and the characteristics of both genes and proteins within this family. Furthermore, a detailed analysis of their transcript expression patterns in two contrasting genotypes of pearl millet covering key growth stages is provided, along with the identification of *cis*-regulatory elements derived from their putative promoter sequences. Additionally, genes displaying overlapping expression patterns across six tissues, as well as those specifically expressed in grains, have been identified. By comparing homologous putative promoter sequences of these grain-specific genes between six pearl millet genotypes, variations in sequences and consequent alterations in the *cis*-elements have been elucidated. Targeting these grain-specific genes might hold significant promise for enhancing diverse grain quality parameters of pearl millet, such as addressing rancidity issues, through the application of forward or reverse genetic approaches.

## Results

### Genome-wide identification, chromosomal distribution, nomenclature, gene structure and collinearity analysis of phospholipases

A keyword search using ‘phospholipase’ initially yielded 6,773 hits across 18 different genomes including 11 pearl millet genotypes listed in the Novogene MilletDB. Using this database, *PLs* belonging to the PI583800 genotype were selected which were 65 in number and included both specific and pseudo phospholipases. The protein sequences of these 65 candidates were investigated for the presence/absence of conserved domains. After analyzing the presence of certain conserved domains such as C2 (which is responsible for Ca^+ 2^/lipid-binding), HKD motifs (H-x-K-x(4)-D), AB-hydrolase domain (α/β hydrolase involved in hydrolysis activity along with HKD motifs), X and Y domains and the PH (pleckstrin-homology) domain specifically located at N-terminus of Ca^+ 2^-independent PLs, a total of 44 phospholipases were selected with C2 (16 members) or hydrolysis (28 members) domains for further studies. The remaining 21 sequences lacked any of the reported conserved domains. Among the selected 44 pearl millet PLs, the PLD subfamily consisted of 21 members, 5 members of the PLC type, 14 classified as PLA1 type, and 4 members belonging to the PLA2 type.

**Fig. 1:**
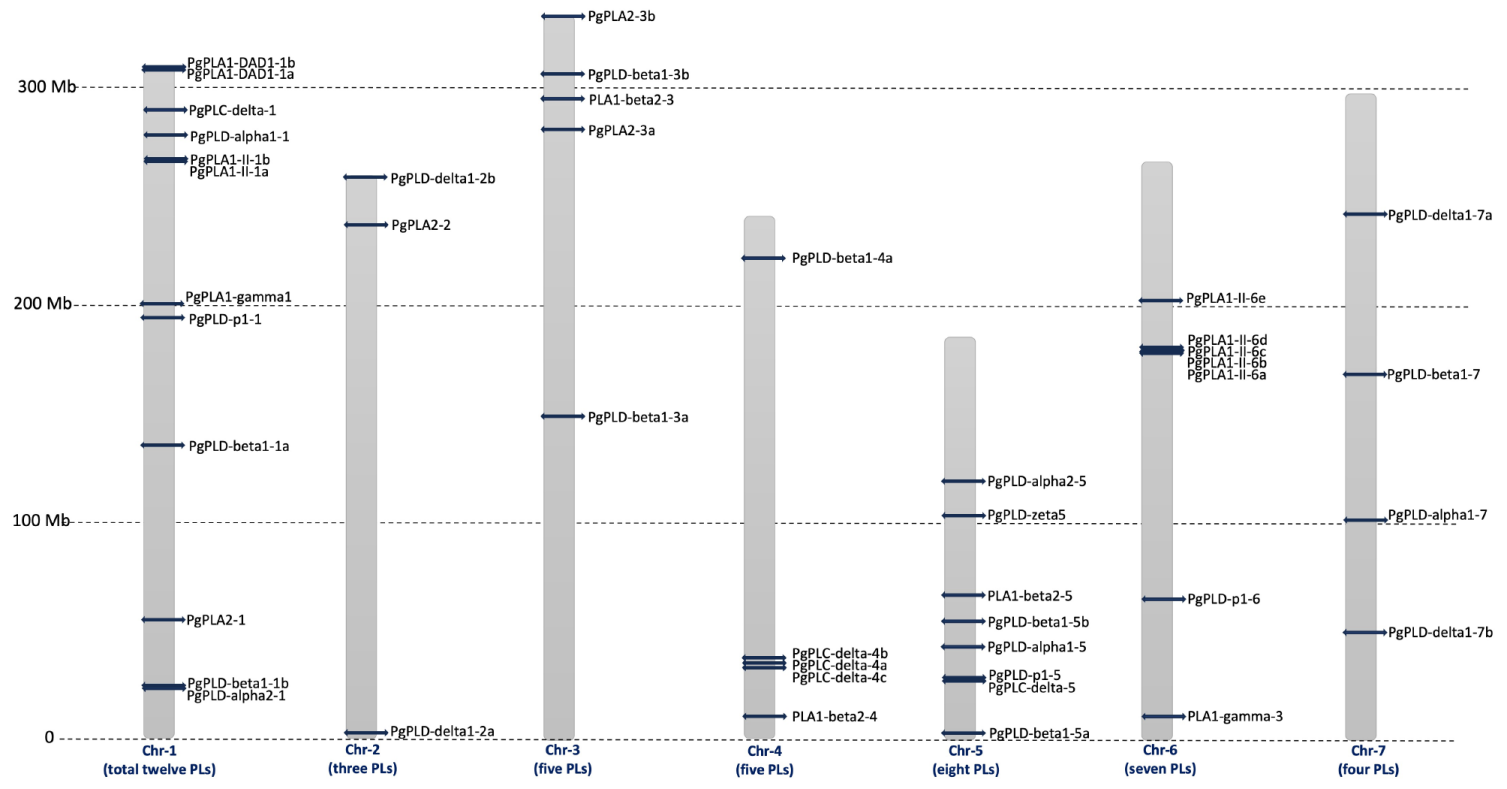
Chromosomal map of pearl millet phospholipase genes. The genome-wide distribution of 44 phospholipases in pearl millet. Each chromosome is accompanied by its respective chromosomal number at the bottom, while the chromosome size is provided on the left side of the map. The number of phospholipase genes per chromosome is denoted within brackets at the bottom of each chromosome. The position of each phospholipase gene on a chromosome is illustrated by horizontal bars across the chromosomes

Phospholipases appeared to be distributed throughout the pearl millet genome with chromosome one carrying the highest number of genes (12) followed by chromosomes five (8), and six (7). Chromosomes three and four harbour 5 genes each, while chromosome seven has 4 and chromosome two has 3 genes (Fig. 1). In rice 39 members of the phospholipase gene family were identified. The distribution of *OsPLs* across the twelve chromosomes of rice revealed that chr-3 contains the highest number, totalling eight, followed by chr-5 with five *OsPLs*. Additionally, chr-6, 9, and 11 each accommodate four *OsPLs*, while chr-1 and 7 house three *OsPLs*. Furthermore, Chr-2, 4, and 8 harbor two *OsPLs*, whereas chr-10 and 12 possess one *OsPL* each (SFig. 1).

The classification of PgPLs was established utilizing information from the MilletDB. In cases where specific isoforms were unlisted in the MilletDB, they were designated according to the respective chromosome and their corresponding position on said chromosome. For instance, within the phospholipase-D subfamily, there exist twenty-one members. Among these, eight were identified as PLD-beta-1, four as delta, three as alpha-1, three as p1, two as alpha-2, and one as zeta isoforms. In the MilletDB repository, the three alpha-1 isoforms (PMD1G06693, PMD5G02305, and PMD7G03361) were simply denoted as PLD-alpha-1. By considering their chromosomal location and order along each chromosome, we assigned them the designations *PgPLD-alpha1-1, PLD-alpha1-5*, and *PgPLD-alpha1-7* due to their presence on chromosomes 1, 5, and 7, respectively. Similarly, two instances of PLD-alpha-2 were titled *PgPLD-alpha2-1* and *PgPLD-alpha-2-5* based on their positioning on chromosomes 2 and 5, respectively.

Amongst the eight beta-1 isoforms present, two resided on chromosomes 1, 3, and 4 each, while one was situated on chromosome 7. Those located specifically on chromosomes 1, 3, and 4 were named *PgPLD-beta1-1a* and *PgPLD-beta1-1b*, respectively, for chromosome 1; *PgPLD-beta1-3a* and *3b* for chromosome 3; and *PgPLD-beta1-4a* and *4b* for chromosome 4. These designations were allocated following their sequential order of occurrence along each respective chromosome. In the A2 category of phospholipases, specifically, five members are identified as PgPLA2 enzymes. Among these, *PgPLA2-1* and *PgPLA2-2* isoforms were named so as they are situated on chromosomes 1 and 2, respectively. Within the PgPLA2 subgroup, three distinct entities were mapped to chromosome 3, denoted as *PgPLA2-3a*, *PgPLA2-3b*, and *PgPLA2-3c* based on their chromosomal locations. Furthermore, within the PgPLAI-II group, a total of seven members were recognized; two were located on chromosome 1 and designated as *PgPLAI-II-1a* and *PgPLAI-II-1-1b*, while the remaining five were positioned on chromosome 6 and named *PgPLAI-II-6a* through *PgPLAI-II-6e*.

**Fig. 2:**
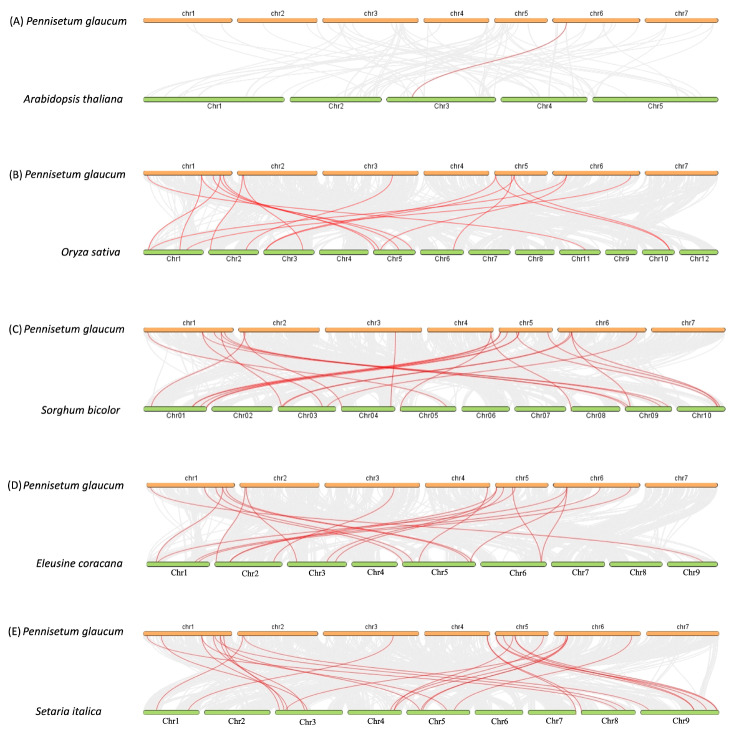
Collinearity relationship of phospholipase genes between pearl millet and other plant species. Synteny analysis was conducted to examine the evolutionary relationship between phospholipase genes in pearl millet and several plants, including (**A**) Arabidopsis (*Arabidopsis thaliana*, 2n = 10), (**B**) rice (*Oryza sativa*, 2n = 24), (**C**) sorghum (*Sorghum bicolour*, 2n = 20), (**D**) finger millet (*Eleusine coracana*, 2n = 36), and (**E**) foxtail millet (*Setaria italica*, 2n = 18). The syntenic blocks between pearl millet and these species are represented by light grey lines in the background, while the collinear phospholipase gene pairs are indicated by red lines. The synteny plot was generated using the step MCscanX option in the TB-tools

To further explore the collinearity relationship among phospholipases in six plant species, the syntenic maps were analyzed to compare PgPLs with their homologs. The number of homologous pairs of phospholipases between pearl millet and various species, including rice, sorghum, finger millet, and foxtail millet, were found to be 17, 22, 19, and 26, respectively. These results indicate a remarkably close genetic relationship between PLs of pearl millet and those of foxtail millet and sorghum, followed by finger millet and rice, suggesting the conservation of the phospholipase gene family during the evolutionary process. A unique collinear pair was identified between a PLD member of pearl millet (PgPLD-beta1-3a) and sorghum (Sobic.004G016900). Interestingly, only one PgPL (PgPLA1-II-6e) from the PLA1 subfamily showed collinearity with Arabidopsis PLA1 (AT3G61680), suggesting a distant phylogenetic relationship between these two species (Fig. 2).

The 44 *PgPLs* were analyzed to investigate the distribution and characteristics of introns and exons, as well as the GC contents of both the gene and corresponding cDNA sequences. The number of introns ranged from 0 to 19. Notably, *PgPLD-p1-1* exhibited the highest count of introns with 19, whereas *PgPLA1-gamma-1*, *PgPLD-alpha2-1*, *PgPLA1-II-1b*, and *PgPLD-alpha1-1* each possessed only one intron. Twenty-four phospholipase genes lacked introns. Genes such as *PgPLA1-DAD1-1b, PgPLD-delta1-2a, PgPLA1-II-1a, PgPLA2-2, PLA1-beta2-3, PLA1-beta2-4, PgPLC-delta-5, PgPLA1-II-6a*, and *PgPLA1-II-6b* displayed zero introns along with a high GC content exceeding 70%. In contrast, *PgPLD-p1-1* is characterized by 19 introns and 21 exons. Additionally, genes like *PgPLD-beta1-5b, PgPLD-beta1-5a*, and *PgPLD-delta1-7b* were noted for having 9 introns and 10 exons each. All of the *PgPLD-alpha* isoforms contain at least one intron. Detailed information regarding the gene structure of individual genes is provided in Supplementary Table 1.

### Protein properties, phylogeny, secondary structure, and regulatory network analyses of phospholipases

Among the 44 PgPLs, PgPLD-p1-1 is the largest with a molecular mass of 129 kDa, followed by PgPLD-beta1-5b (116 kDa) and PgPLD-delta1-2a (100 kDa). The remaining PLs have a molecular mass of less than 100 kDa. All these proteins have similar isoelectric points ranging from 5.47 to 9.41. This might be to reduce columbic repulsions as these proteins have interactive properties. The GRAVY indices, which are an indicator of the hydrophobicity of a peptide, are < 0. Peptides with hydropathy scores < 0 are highly hydrophilic and tend to form flexible structures with other molecules [33, 34]. The secondary structure of PLs exhibits varying proportions of α, β, and distorted regions. For example, PgPLA2-1 displays the highest percentages of α-helical and distorted structures at 40% and 34%, respectively, whereas PgPLD-beta1-4a shows the highest β-sheet content at 53%. PgPLC-delta-4a and 4b possess an equal distribution of all three structural types, amounting to 19%. Across all phospholipases, there are regions of disordered protein segments present, ranging from as little as 1% in PgPLA2-3a to as high as 40% in PgPLA2-1. These disordered regions are instrumental in facilitating the organization of signalling complexes through reversible protein-protein interactions that support the formation of reversible cellular assemblies and contribute to the development of stable amyloid frameworks [35, 36]. Because PLs are highly interactive, the presence of more α- helical and distorted contents reinforces their activity. While a majority of PLs have Ca^+ 2^ as the preferred metal-binding site, certain phospholipases like PLA1-beta2-4, PgPLC-delta-1, PgPLC-delta-4a, PgPLC-delta-4b, PgPLD-p1-6, and PgPLD-p1-5 have been identified with La^+ 3^ as potential metal-binding site. Additionally, PgPLC-delta-4c has been found to potentially bind Mn^+ 2^. Furthermore, acetate, sulfate, and phosphate act as putative non-metal cofactors for phospholipases. The properties of phospholipases such as size, *p*I, the percentage of α-helices and β-sheets, GRAVY indices, and the type of metal/non-metal ligands are detailed in Table 1.

**Table 1:** A comprehensive analysis of the *cis*-regulatory elements found in the promoters of phospholipase genes in pearl millet. The presence of each *cis*-element in the putative promoter region is denoted by an asterisk (*), indicating the number of copies

Gene ID	Protein code	TGA-element	DRE core	STRE	W-Box	WRE3	as-1	ABRE	GATA motif	MYC	MYB	LTR	TCA	SP1	ERE	*P*-box	RY	OCT	ARE	WUN Motif	O2 site	GARE	TC-rich repeats
**PMD3G08871**	**PgPLA2-3b**	*	*	*	*	*	*				***												
**PMD5G02305**	**PgPLD-alpha1-5**	*	*	****				*	*	*	**	*	*										
**PMD1G06693**	**PgPLD-alpha1-1**	****		*****			****	***		**	*	*		**									
**PMD6G04197**	**PgPLA1-II-6b**					**		***		*	***												
**PMD1G08121**	**PgPLA1-DAD1-1b**	*		***			*	****	*	*	**				*								
**PMD1G08120**	**PgPLA1-DAD1-1a**	*	*	**		*	*	****		**	******			*		*							
**PMD3G06955**	**PgPLA2-3a**	**			*	*	**	*******		***	*****			*			*	*					
**PMD2G06735**	**PgPLA2-2**	**	**	***			**	*		**	*****			***					*				
**PMD3G07336**	**PLA1-beta2-3**	*	**	***	*		*			*	***	*		***				*					
**PMD1G07144**	**PgPLC-1**	**		***		*	**	****		**	**************					*							
**PMD5G04890**	**PgPLD-alpha2-5**	*					*	*			*			**				*		*			
**PMD1G06220**	**PgPLA1-II-1b**	*		**	*		*	**		**	***********								**				
**PMD1G04489**	**PgPLA1-gamma1**	**	*	***	*		**	****			****			*					**		**		
**PMD1G06219**	**PgPLA1-II-1a**	**		*			**	***		**	**********	*							*				
**PMD6G04193**	**PgPLA1-II-6a**			*		**				*	*****				*				***			*	
**PMD6G04201**	**PgPLA1-II-6d**	*					*	***		*	**				*			*	*				
**PMD6G04790**	**PgPLA1-II-6e**	*	**			*		****		**	****				**				**		**		
**PMD5G04439**	**PgPLD-zeta5**	**		**	*		**	*		****	***			**				*	*				
**PMD1G04280**	**PgPLD-p1-1**	***			*	**	***	***		*	**			**					**				**
**PMD5G03093**	**PLA1-beta2-5**	*		**			*	******			*								*		*		
**PMD6G02138**	**PgPLD-p1-6**	*		***		*	*	****			*****			*					***				
**PMD4G01436**	**PgPLC-4c**		**	**				*******		*	*****			*									*
**PMD5G01606**	**PgPLC-5a**	*		***		*	*	****			*****			*					***				
**PMD1G01583**	**PgPLA2-1**	***			**		***	*		****	*********												*
**PMD4G01333**	**PgPLC-4a**	**		*	**	*	**	***			**					*			*				
**PMD4G01357**	**PgPLC-4b**	*		****	**		*			*				*					***	*	*		
**PMD1G00695**	**PgPLD-beta1-1b**	*****	*	**		*	*****	***		*	****			*	**				*	*	*		
**PMD4G05665**	**PgPLD-beta1-4a**	****		***			****			**	**	*		**					***	*	*		
**PMD6G00496**	**PLA1-gamma-3**			**	*		*				******	*				*	*			*		*	*
**PMD4G00298**	**PLA1-beta2-4**		*	**	*	**	**	*		*	*******			*		*			*	*	*		
**PMD5G00144**	**PgPLD-beta1-5a**	*******	***	***		*	*******	******			*	*				*							
**PMD5G01607**	**PgPLD-p1-5**	*		*		*	*			***	*		******										*
**PMD7G05807**	**PgPLD-delta1-7a**			*****															***				
**PMD7G02029**	**PgPLD-delta1-7b**		**	****		******		***			***			*									
**PMD3G07688**	**PgPLD-beta1-3b**	****		***			****			*	**	*		**					***	*	*		
**PMD7G04652**	**PgPLD-beta1-7**	****		***			****			*	**	*		**					***	*	*		
**PMD5G02728**	**PgPLD-beta1-5b**	*		**	*	*	*	*		**	****	*		*					*				
**PMD2G07986**	**PgPLD-delta1-2b**	**		*****			**	*		**	************		**	**		*	*					*	
**PMD3G04495**	**PgPLD-beta1-3a**	****		***			****			*	*	*		**					***	*	*		
**PMD2G00129**	**PgPLD-delta1-2a**	*	**		*	*	*			**	***	*		*							*		
**PMD1G03427**	**PgPLD-beta1-1a**	****		****			****			*	*	*		**					***	*	*		
**PMD6G04200**	**PgPLA1-II-6c**	*	*	*			*			***	******					*					**		
**PMD1G00694**	**PgPLD-alpha2-1**	*					*	**			*				*				*				
**PMD7G03361**	**PgPLD-alpha1-7**	*					**	*		***	*										*		

**Fig. 3:**
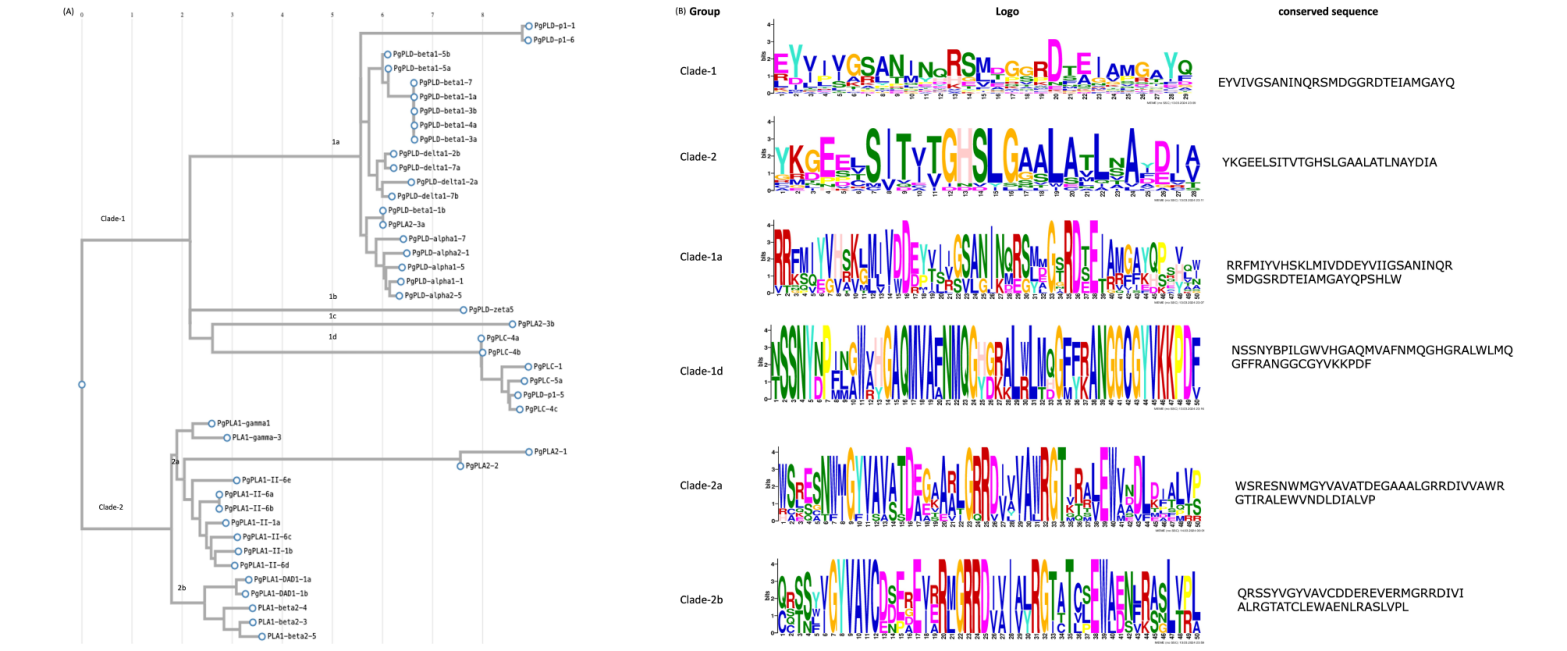
Phylogeny and motif analysis of phospholipase proteins. (**A**) The alignment of 44 phospholipase protein sequences was performed using ClustalW, followed by the construction of a phylogenetic tree to investigate their evolutionary relationships. (**B**) Subsequently, the sequences of phospholipases belonging to the same clades were subjected to MEME Suite 5.5.5 to identify conserved sequences within each group. These conserved sequences were further analyzed by submitting them to the NCBI-conserved domain database to identify the resulting motif

To assess the evolutionary relationships within the PL protein family, an unrooted phylogenetic tree was developed utilizing the full-length amino acid sequence and a ‘neighbour joining algorithm’ with a bootstrap value set at 1000. The resulting tree exhibited two distinct clades. Clade-1 was identified as encompassing PLD and PLC isoforms (Fig. 3). PLD enzymes characterized by a conserved HKD motif domain function as active bi-lobed monomers, where each monomer comprises two domains, each housing one instance of the HKD motif. The amalgamation of the two HKD motifs from these domains produces a singular active site [37]. PLDs operate through a standard two-step ping-pong catalytic process that involves an enzyme-substrate intermediate for cleaving phosphodiester bonds [45]. The participation of two histidine residues from the HKD motifs is pivotal in this catalytic mechanism [38]. Clade-2 consists of PL-A1 and A2 proteins falling under the superfamily alpha/beta hydrolases category [39]. Their catalytic mechanism typically involves a triad of three residues (serine, glutamate or aspartate, and a histidine) with nucleophilic attack on a carbonyl carbon atom often being part of the process [40].

Further classification revealed that Clade-1 could be subdivided into four sub-clades: clade-1a, 1b, 1c, and 1d; while Clade-2 was segmented into clade-2a and 2b. Clade-1a and 1b house all PLD isoforms; clade-1d contains both PgPLD and PgPLC isoforms; whereas clade-1b encompasses a PgPLA2 member. On the other hand, clade-2a features PgPLA1-gamma isoforms, while clade-2b includes PgPLA1-beta, DAD1, and A1-II isoforms. Clade-1a proteins have a regulatory C2 domain, while clade-1d group proteins exhibit calcium-dependent PLC catalytic properties. Clade-2a contains PLAII, A1-gamma, and PLA1-II proteins. PLA1s are characterized by the presence of a GXSXG motif and the presence of a catalytic triad, which is composed of a serine, an aspartic acid, and a histidine residue. This catalytic triad is buried inside the structure. The active site of these proteins is shielded by a lid, which, in the process of interfacial activation, opens up, granting access to the lipid substrate to interact with the active site [24, 25]. The Clade-2b group comprises two proteins, PgPLA1-beta and PgPLDAD1. In addition, a comparative analysis was performed between Os and PgPLs by constructing an integrated phylogenetic tree incorporating the phospholipase protein sequences of rice and pearl millet (SFig.2).

The network of regulatory associations among phospholipases was established through the submission of candidate proteins to the STRING database, a tool that constructs networks based on functional associations (indirect interactions) and includes direct physical interactions. Among the 44 proteins submitted, 34 were successfully identified by the database. The subsequent interaction analyses focused on these 34 protein nodes, revealing 92 edges with an average node degree of 5.41, an average local clustering coefficient of 0.471, and a protein-protein interaction enrichment p-value of 1.0e-16. The notably higher number of edges (92) in comparison to the expected value (0) indicates a significantly increased level of interactions or connections. This enrichment strongly implies that the proteins are interconnected, at least to some extent, on a biological level, as a cohesive group. Notably, PgPLD-alpha2-5 and PgPLD-delta1-7b were observed to interact with nine other PLD nodes and one PLA1 node each, while PgPLD-alpha1-7 interacted with eight PLD nodes, and PgPLD-alpha1-1 engaged with seven PLD nodes and one PLA1 node in this network analysis (SFig. 3).

### Tissue-specific expression of phospholipases

**Fig. 4:**
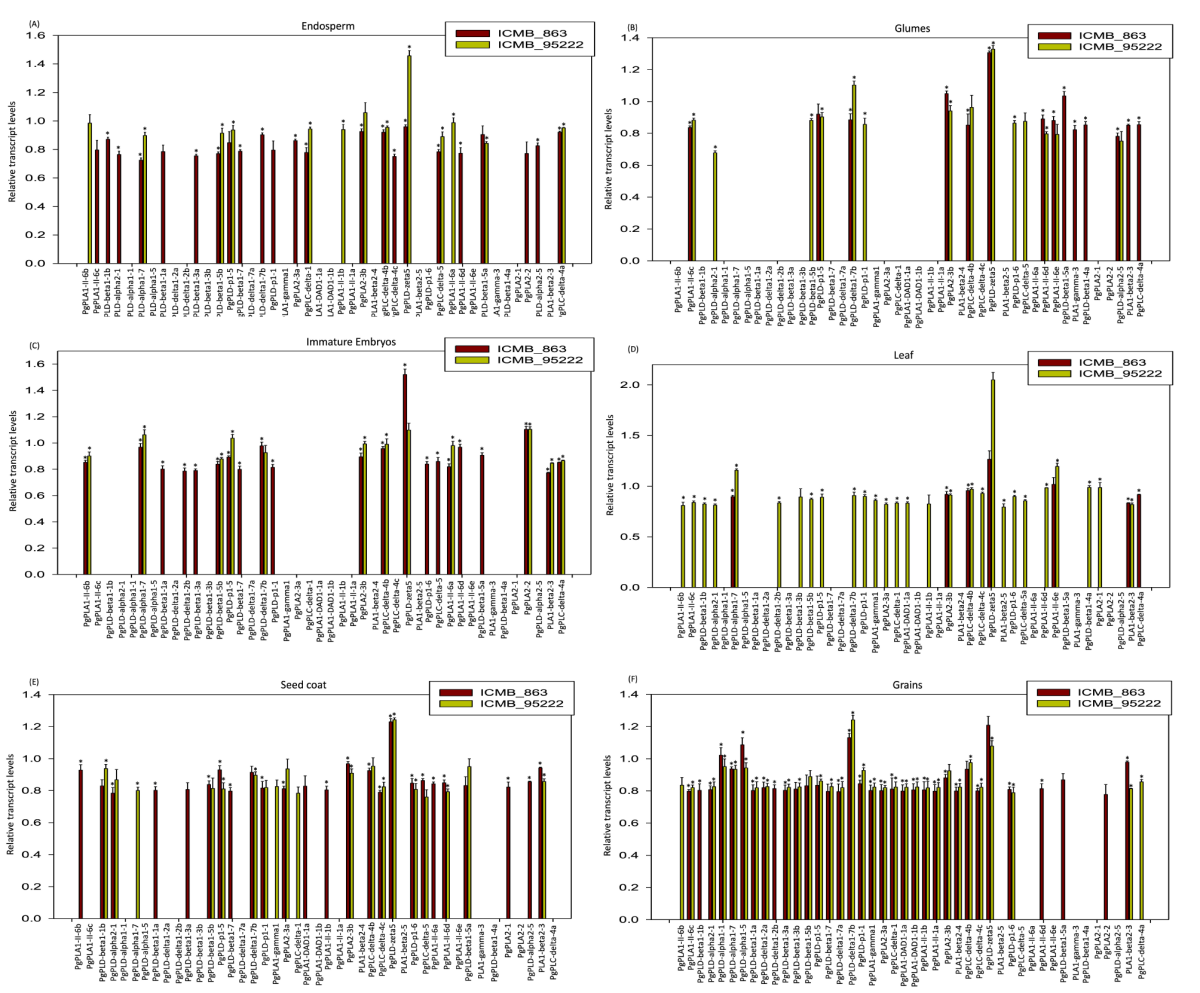
Expression analysis of 44 phospholipases in six different tissues across two contrasting genotypes of pearl millet. The native transcript levels of 44 phospholipases were investigated across six different tissues in two pearl millet genotypes using qRT-PCR. The tissues examined included (**A**) endosperm, (**B**) glumes, (**C**) immature embryos, (**D**) leaves, (**E**) seed coat, and (**F**) grains. The eukaryotic initiation factor 4 F (*eIF4F*) was utilized as an internal reference gene for normalization. Statistical significance was determined using a one-way ANOVA analysis conducted through SigmaPlot v.11, with asterisks denoting significance at *P* < 0.05 in the bar diagrams

Quantitative real-time PCR (qRT-PCR) was performed to study the transcript expression patterns of the phospholipase gene family. This analysis was carried out on six different tissues from two contrasting genotypes of pearl millet for rancidity (SFig. 4). The primers utilized in this study for transcript analysis have been provided in Supplementary Table 2. Within the high rancid genotype (ICMB-863), the highest number of genes were expressed in grains (34 genes), followed by seed coat (25), endosperm (23), immature embryos (21), and glumes (14), while leaves showed expression of only seven genes. Among the 34 genes expressed in grains of the ICMB-863 genotype, nine (*PgPLA1-II-1a, PgPLD-alpha1-5, PgPLD-delta1-7a, PgPLD-alpha1-1, PLA1-beta2-4, PgPLA1-DAD1-1b, PgPLD-beta1-3b, PgPLD-delta1-2a*, and *PgPLA1-gamma1*) were specific to grains. Furthermore, three genes (*PgPLD-zeta5, PgPLA2-3b*, and *PgPLC-delta-4b*) were found to be expressed in all six tissues. Notably, four genes (*PgPLD-delta1-7b, PgPLD-beta1-5a, PgPLD-p1-5*, and *PgPLA1-II-6d*) were commonly expressed among endosperm, glumes, grains, immature embryos, and seed coat (Fig. 4). Interestingly, glumes demonstrated tissue-specific expression of two genes, *PgPLA1-gamma-3* and *PgPLD-beta 4a*. The remaining genes exhibited varying degrees of overlap in their expression across two to five tissues.

The *PLA1-beta2-3* gene exhibited expression in glumes, grains, immature embryos, leaves, and seed coat. In contrast, *PgPLC-delta-4a* showed expression in the endosperm, glumes, immature embryos, leaf, and seed coat. The *PgPLD-alpha1-7* gene was found to be expressed in endosperm, grains, immature embryos, and leaves. Similarly, *PgPLD-p1-6* displayed expression in grains, immature embryos, and the seed coat. Notably, *PgPLA1-II-6c* was observed to be expressed in endosperm, glumes, and grains. Moreover, *PgPLC-delta-5* exhibited upregulation specifically in the endosperm, immature embryos, and seed coat. Additionally, the genes *PgPLD-delta1-2b* and *PgPLC-delta-1* were expressed in grains and immature embryos, while the expression of *PgPLD-alpha2-5* was detected in the endosperm and glumes. Furthermore, *PgPLA1-II-6e* showed expression in glumes and leaf; whereas both *PgPLA1-DAD1-1a* and *PgPLA1-II-1b* were expressed in grains and seed coat. Likewise, *PgPLA1-II-6b* and *PgPLA1-II-6a* exhibited expression specifically in immature embryos and seed coat (Fig. 4).

In the low-rancid genotype (ICMB-95222), the highest number of *PLs* was found to be expressed in grains, totalling 31 genes. Interestingly, in contrast to ICMB-863, a significant number of genes were expressed in the leaf (28 genes), followed by the seed coat (19 genes), glumes (14 genes), endosperm (13 genes), and immature embryos (12 genes). Among the 31 *PLs* expressed in grains, ten (*PgPLA1-II-1a, PgPLD-alpha1-5, PgPLD-delta1-7a, PgPLD-alpha1-1, PLA1-beta2-4, PgPLD-beta1-3a, PgPLA1-DAD1-1b, PgPLD-delta1-2a, PgPLD-beta1-7*, and *PgPLD-beta1-1a*) were identified as specific. Additionally, five genes (*PgPLD-beta1-5b, PgPLD-p1-5, PgPLD-zeta5, PgPLA2-3b*, and *PgPLC-delta-4b*) showed common expression across all six tissues. Furthermore, four *PL* genes, *PgPLA2-1, PgPLA1-beta2-5*, *PgPLD-beta1-4a*, and *PgPLD-delta1-2b* exhibited tissue-specific expression in leaves. *PgPLD-alpha2-5* and *PgPLA2-2* were specifically expressed in glumes and immature embryos, respectively (Fig. 4).

The expression of *PgPLD-delta1-7b* was identified in glumes, grains, immature embryos, leaves, and seed coat; *PgPLD-alpha1-7* was detected in endosperm, grains, immature embryos, leaves, and seed coat; while *PgPLC-delta-4a* exhibited expression in endosperm, grains, immature embryos, and seed coat. Additionally, *PgPLA1-II-6b* manifested expression in endosperm, grains, immature embryos, and leaves. *PgPLC-delta-1* was observed to be expressed in endosperm, grains, leaves, and seed coat; whereas *PgPLC-delta-5* displayed expression in endosperm, glumes, leaves, and seed coat. *PLA1-beta2-3* showed upregulation in grains, immature embryos, and leaves; *PgPLA1-II-1b* was upregulated in endosperm, grains, and leaves, whereas the expression of *PgPLA1-II-6c* was noted in glumes, grains, and leaves. Furthermore, *PgPLA1-II-6d* was found to be expressed in glumes, leaves, and seed coat. *PgPLA1-II-6a* was found to be expressed in the endosperm and immature embryos, while transcripts of *PgPLA1-II-6e* were upregulated in glumes and leaves. Furthermore, *PgPLD-beta1-5a* demonstrated expression in both endosperm and seed coat, whereas *PgPLD-beta1-1b* exhibited expression in leaves and seed coat. Additionally, *PgPLD-p1-6, PgPLD-p1-1*, and *PgPLD-alpha2-1* were upregulated in glumes, grains, leaves, and seed coat. On the other hand, *PgPLA2-3a, PgPLA1-gamma1*, and *PgPLC-delta-4c* were found to be expressed in grains, leaves, and seed coats. The expression of *PgPLA1-DAD1-1a* and *PgPLD-beta1-3b* was detected in grains and leaves, respectively. Three phospholipases, *PgPLA2-3b, PgPLC-delta-4b*, and *PgPLD-zeta5* were expressed in all the tissues in both genotypes.

The expression data was also examined through a comparative analysis between two genotypes, ICMB-863 and ICMB-95222. Notably, among the 30 PL genes demonstrating expression in grains across both genotypes studied, *PgPLD-alpha1-1, PgPLD-alpha1-5, PgPLD-delta1-7a, PgPLA1-II-1a*, and *PgPLD-delta1-2a* were exclusively detected in the grains of two genotypes. Conversely, the remaining 25 candidates exhibited overlapping expression patterns between two or more tissues. Moreover, within this subset of five genes, comparatively high expression levels were observed for *PgPLD-alpha1-1* and *PgPLD-alpha1-5* in ICMB-863, a high rancid pearl millet genotype. Furthermore, in immature embryos, glumes, endosperm, seed coat, and leaves, there were 13, 10, 11, 17, and 7 genes expressed commonly in the respective tissues of both genotypes.

Of particular note is the exclusive expression of *PgPLD-alpha1-1, PgPLD-alpha1-5, PgPLD-delta1-7a, PgPLA1-II-1a*, and *PgPLD-delta1-2a* in grains of both genotypes without any detectable expression in the other five tissues. The overlap in the expression pattern of 44 phospholipase genes in six tissues of high and low rancid genotypes was represented as Venn diagrams (SFig. 5). Supplementary Table 3 provides a detailed list of phospholipases that exhibited specificity or an overlap in the expression in each tissue of two genotypes. In addition, SFig. 6 provides a flowchart that portrays the sequential steps and tools employed for the identification and analysis of the phospholipase gene family in pearl millet.

### *In silico* analysis of putative promoter regions of pearl millet phospholipase genes

**Fig. 5:**
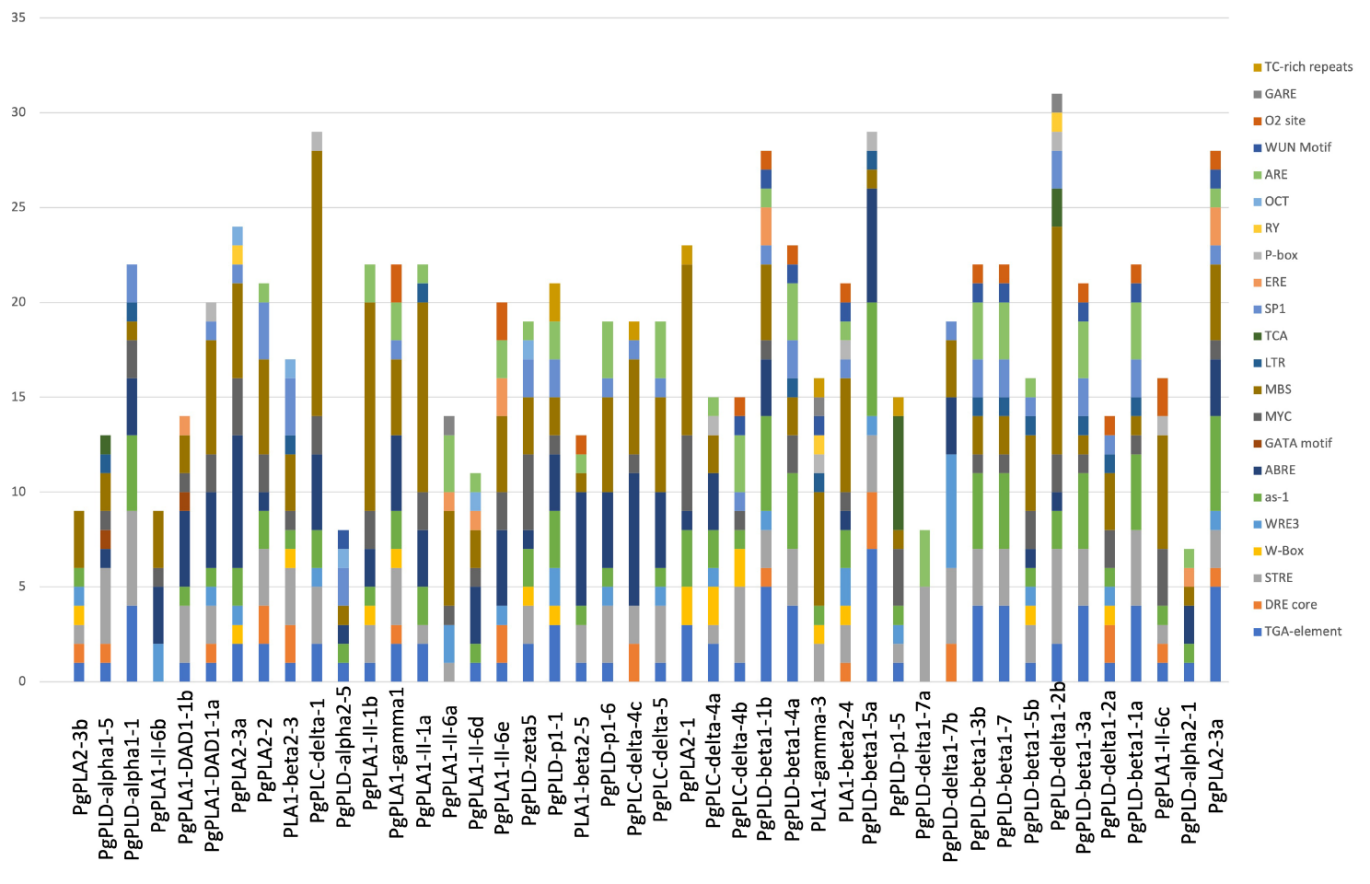
*In silico* analysis of 1 kb upstream region of pearl millet phospholipase genes for the identification of *cis*-regulatory elements. The 1 kb upstream putative promoter sequences of phospholipase genes exhibit multiple copies of different *cis*-elements. The bar diagrams visually illustrate the enrichment of distinct stress/signal-responsive elements, with each colour denoting a specific element highlighted on the right side

The analysis of the 1 kb upstream sequence resulted in the identification of multiple stress and signal-responsive elements in the putative promoter regions of all the 44 *PgPLs*. Abiotic stress-responsive elements that are associated with oxidative stress such as STRE (STress-Response Element), as-1 (*activation sequence-1*, an oxidative-stress responsive element [41]), and hypoxia (O_2_-elements) and cold temperatures like LTR (Low-Temperature Response) and MYC, are present in multiple copies. In addition, dehydration stress-responsive elements such as DRE-core (dehydration-responsive element) containing the core sequence (A/GCCGAC), and MBS (Myb Binding Site) are widely distributed within the putative promoter regions of *PL* genes. MBS is a binding site for MYB-related transcription factors that are involved in the regulation of genes responsive to water-deficit conditions [42]. W-box elements which serve as a binding site for WRKY transcription factor members [43] are also found in many copies. The presence of these elements in the promoter regions suggests that the corresponding genes become activated under water stress or drought conditions. In addition to abiotic stresses, elements that respond to hormones such as abscisic acid (ABRE-Abscisic acid responsive element), estrogen (ERE-element), methyl jasmonate (TGACG-motif), salicylic acid (TCA-motif), gibberellic acid (GARE-Gibberellic acid responsive element), and auxin (TGA-motif and AuxR-Auxin responsiveness) are also present in multiple copies (Fig. 5). Motifs that regulate the transcription such as Sp1, and Pribnow box (P-box) are also present. Elements that respond to biotic stimuli such as WUN-motif, WRE-3 element, and TCA-element are also distributed widely (Fig. 5).

Among 44 phospholipase promoters analyzed, the presence of multiple copies of the MBS motif was observed in all except for *PgPLC-delta-4b* and *PgPLD-delta1-7a*. Notably, the highest occurrences of MBS repeats were identified in *PgPLC-delta-1* (14 repeats), followed by *PgPLD-delta1-2b* (12), *PgPLA1-II-1b* (11), and *PgPLA1-II-1a* (10). Furthermore, PgPLA2-3b exhibited three MBS repeats along with additional motifs such as TGA, DRE, STRE, WRE-3, as-1, and W-box. Among the genes that were specifically expressed in grains, *PgPLD-alpha1-1* displayed five STRE repeats, four TGA repeats, as well as one occurrence each of ABRE, MYC, Sp-1, and LTR motifs. Similarly, *PgPLD-alpha1-5* contained four STRE repeats alongside two MBS repeats and single instances of TCA, LTR, MYC, GATA, ABRE, and TGA motifs. Noteworthy is that *PgPLD-delta1-7a* encompassed five STRE elements combined with three ARE elements. Moreover, the promoter region of *PgPLA1-II-1a* featured ten MBS repetitions in addition to three ABRE occurrences and two each of *as-1* and TGA motifs; it also contained singular instances of STRE, ARE, and LTR motifs. Additionally noted were three MBS repetitions in the promoter of *PgPLD-delta1-2a* together with two MYC and DRE motifs; this promoter also displayed one instance each of TGA, WRE-3, as-1, LTR, SP1, O2 and W-box elements.

The TC-rich repeats, which play a role in defence mechanisms and response to biotic stress [44], were found within the promoter sequences of *PgPLD-p1-1*, *PgPLC-delta-4c*, *PgPLA2-1*, *PLA1-gamma-3*, and *PgPLD-p1-5*. Moreover, W-box motifs, serving as binding sites for stress-responsive WRKY transcription factors [45], are present as multiple copies in the promoters of *PgPLA2-1*, *PgPLC-delta-4a*, and *PgPLC-delta-4b*. Additionally, they appear as a single instance in the promoter sequences of *PgPLD-p1-1*, *PgPLD-zeta5*, *PgPLA1-gamma1*, *PgPLA1-II-1b*, *PLA1-beta2-3*, *PgPLA2-3a*, and *PgPLA2-3b*. Furthermore, the WUN motif associated with wounding stress responses [46] is detected in a single copy in several sequences, including *PgPLD-alpha2-5, PgPLC-delta-4b, PgPLD-beta1-1b, PgPLD-beta1-4a, PgPLA1-gamma-3, PgPLA1-beta2-4, PgPLD-beta1-3b, PgPLD-beta1-7, Pg PLD-beta 13a, Pg PLDbeta11a*, and *PgPLDA23a*. TCA-element that plays a key regulatory role in salicylic acid-induced MYB15 activation [47] is present as six copies in *PgPLD-p1-5*, two copies in *PgPLD-delta1-2b*, and a single motif in *PgPLD-alpha1-5*. The *as-1* which is activated by reactive oxygen species is present in 38 out of 44 phospholipase promoters. Table 2 presents a detailed analysis of *cis*-regulatory elements and their repeats in the putative promoter regions of phospholipase genes.

**Table 2:** Properties of phospholipase proteins

GeneID	Protein name	protein length	Mol Wt	*p*I	GRAVY	rich aminoacid (%)	disordered region	alpha helix	beta strand	Non-metal ligand	Metal ligand
**PMD1G03427.1**	**PgPLD-beta1-1a**	343	37	7.7	-0.146	Val	14	8	41	Phosphite ion	calcium
**PMD1G04280.1**	**PgPLD-p1-1**	1133	129	6.82	-0.485	Leu (10%)	19	20	14	Phosphite ion	
**PMD1G04489.1**	**PgPLA1-gamma1**	604	68	6.43	-0.687	Ala (11%)	28	22	8	Sulfate ion	
**PMD1G00695.1**	**PgPLA2-3a**	157	17	5.67	-0.075	Gly and Leu (11%) each)	1	22	17	Phosphite ion	calcium
**PMD1G00694.1**	**PgPLD-alpha2-1**	679	74	6.48	-0.266	Gly (9%)	9	13	23	Phosphite ion	calcium
**PMD1G01583.1**	**PgPLA2-1**	181	19	7.52	-0.09	Ala (14%)	34	40	0		calcium
**PMD1G07144.1**	**PgPLC-delta-1**	534	60	6.02	-0.565	Leu (8%)	24	14	21	Acetate ion	Lanthanum (III) ion
**PMD1G08120.1**	**PgPLA1-DAD1-1a**	444	48	5.74	-0.236	Leu (10%)	7	27	16	Sulfate ion	
**PMD1G08121.1**	**PgPLA1-DAD1-1b**	403	42	5.47	-0.23	Ser (12%)	14	21	13	Sulfate ion	
**PMD2G00129.1**	**PgPLD-delta1-2a**	913	100	7.97	-0.383	Ala (11%)	11	18	15		calcium
**PMD1G06220.1**	**PgPLA1-II-1b**	405	43	5.52	-0.05	Ala (13%)	10	19	13		
**PMD1G06219.1**	**PgPLA1-II-1a**	412	44	6.4	-0.25	Ala (15%)	9	21	10		
**PMD1G06693.1**	**PgPLD-alpha1-1**	820	91	5.92	-0.342	Ala (8%)	4	15	22	Phosphite ion	calcium
**PMD2G06735.1**	**PgPLA2-2**	157	16	5.47	0.165	Ala (13%)	23	43	0		calcium
**PMD2G07986.1**	**PgPLD-delta1-2b**	862	96	8.53	-0.353	Val (8%)	9	16	18	Phosphite ion	calcium
**PMD3G04495.1**	**PgPLD-beta1-3a**	343	37	7.16	-0.135	Val (11%)	17	8	38		calcium
**PMD3G06955.1**	**PgPLA2-3a**	158	16	6.85	0.02	Leu (13%)	1	22	17	Sulfate ion	
**PMD3G07336.1**	**PLA1-beta2-3**	536	57	9.36	-0.352	Ala (13%)	31	21	10	Phosphite ion	calcium
**PMD3G07688.1**	**PgPLD-beta1-3b**	458	50	7.95	-0.14	Val (10%)	17	6	38		calcium
**PMD3G08871.1**	**PgPLA2-3b**	149	15	8.7	-0.119	Ala (13%)	26	38	0	Sulfate ion	
**PMD4G00298.1**	**PLA1-beta2-4**	515	54	9.41	-0.138	Ala (15%)	29	24	8	Acetate ion	Lanthanum (III) ion
**PMD4G01333.1**	**PgPLC-delta-4a**	595	66	6.05	-0.447	Leu (9%)	19	19	19	Acetate ion	Lanthanum (III) ion
**PMD4G01357.1**	**PgPLC-delta-4b**	590	66	6.05	-0.434	Leu (9%)	19	19	19	Acetate ion	Lanthanum (III) ion
**PMD4G01436.1**	**PgPLC-delta-4c**	607	68	6.07	-0.576	Leu (9%)	21	17	20		Manganese (ii) ion
**PMD5G04439.1**	**PgPLD-zeta5**	556	62	8.89	-0.305	Ser (9%)	9	20	16	Phosphite ion	calcium
**PMD5G04890.1**	**PgPLD-alpha2-5**	818	91	6.6	-0.297	Gly (9%)	5	16	20	Phosphite ion	calcium
**PMD5G02728.1**	**PgPLD-beta1-5b**	1044	116	7.15	-0.449	Pro (9%)	27	13	17	Sulfate ion	
**PMD5G03093.1**	**PLA1-beta2-5**	556	60	8.92	-0.372	Ala (13%)	27	25	8	Phosphite ion	
**PMD6G02138.1**	**PgPLD-p1-6**	1103	125		-0.448	Leu (8%)	15	20	16	Acetate ion	Lanthanum (III) ion
**PMD5G01607.1**	**PgPLD-p1-5**	210	23	7.01	-0.292	Val (10%)	11	3	41	Acetate ion	Lanthanum (III) ion
**PMD5G01606.1**	**PgPLC-delta-5**	283	32	6.76	-0.339	Leu (10%)	12	32	9	Sulfate ion	
**PMD6G04193.1**	**PgPLA1-II-6a**	428	45	6.5	-0.105	Ala (13%)	11	26	12	Sulfate ion	
**PMD6G04197.1**	**PgPLA1-II-6b**	428	45	6.5	-0.099	Ala (13%)	10	25	11		
**PMD6G04200.1**	**PgPLA1-II-6c**	427	47	5.91	-0.419	Ala (10%)	11	19	12	Sulfate ion	
**PMD6G04201.1**	**PgPLA1-II-6d**	394	44	6.33	-0.343	Leu (9%)	10	19	15		
**PMD6G04790.1**	**PgPLA1-II-6e**	461	49	5.96	-0.059	Ala (11%)	15	18	11	Phosphite ion	calcium
**PMD4G05665.1**	**PgPLD-beta1-4a**	201	22	10.6	-0.688	Arg (15%)	18	0	53	Phosphite ion	calcium
**PMD5G02305.1**	**PgPLD-alpha1-5**	831	92	6.45	-0.391	Ala (9%)	7	15	19	Phosphite ion	calcium
**PMD5G00144.1**	**PgPLD-beta1-5a**	665	75	8.76	-0.524	Arg (7%)	3	20	16	Sulfate ion	
**PMD6G00496.1**	**PLA1-gamma-3**	542	60	9.8	-0.539	Arg (12%)	15	22	10	Phosphite ion	calcium
**PMD7G04652.1**	**PgPLD-beta1-7**	343	37	6.8	-0.124	Val (11%)	15	7	37	Phosphite ion	calcium
**PMD7G05807.1**	**PgPLD-delta1-7a**	872	98	6.7	-0.387	Gly (7%)	10	15	20	Phosphite ion	calcium
**PMD7G03361.1**	**PgPLD-alpha1-7**	826	91	5.48	-0.275	Ala (11%)	5	17	18	Phosphite ion	calcium
**PMD7G02029.1**	**PgPLD-delta1-7b**	855	96	6.8	-0.432	Ala (7%)	9	17	19	Phosphite ion	calcium

**Fig. 6:**
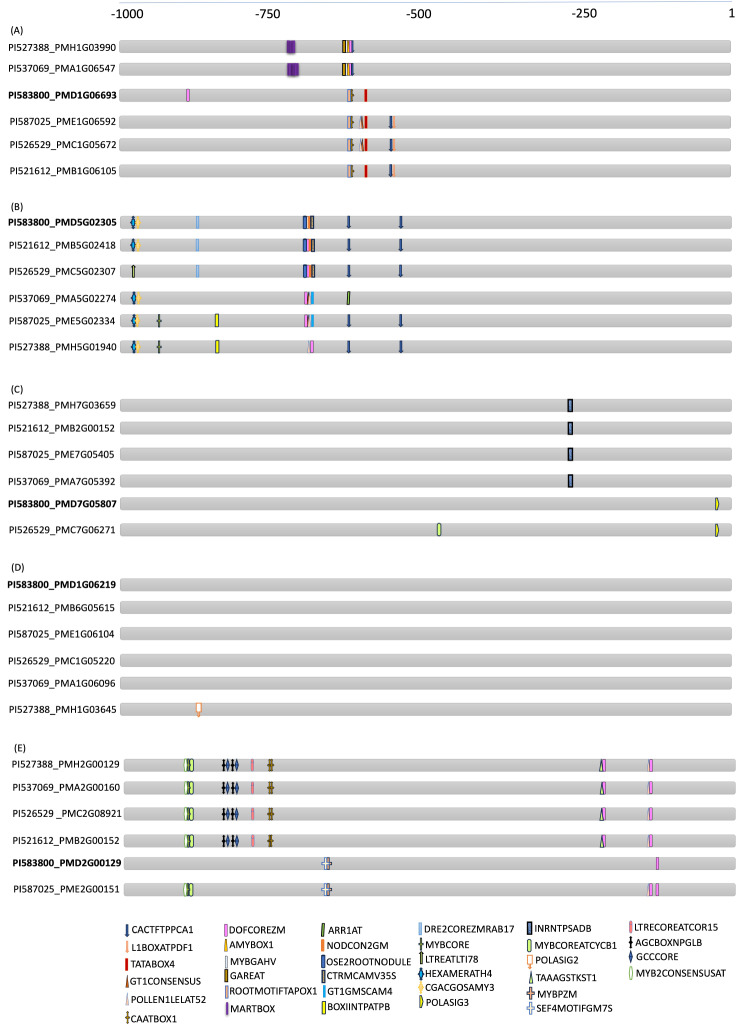
Comparative analysis of putative promoters of five grain-specific genes across six pearl millet genotypes. The *cis*-element analysis was conducted on the promoters of five grain-specific phospholipase genes, namely (**A**) *PgPLD-alpha1-1*, (**B**) *PgPLD-alpha1-5*, (**C**) *PgPLD-delta1-7a*, (**D**) *PgPLA1-II-1a*, and (**E**) *PgPLD-delta1-2a*, along with their corresponding homologous sequences from six different genotypes of pearl millet [107]. The analysis revealed the presence of various variations, such as single-nucleotide polymorphisms, deletions, and insertions within these sequences. These genetic variations were found to have an impact on the corresponding *cis*-regulatory elements, leading to either the addition or deletion of these key elements. Shapes and colours depict *cis*-element as indicated at the bottom of the illustration. A scale positioned at the top of the figure serves to indicate the likely localization of the elements being represented. Provided on the left side are the gene accessions for the corresponding genotype

The alignment of five grain-specific genes with homologous sequences from six different pearl millet genotypes revealed no sequence variation in the CDS region including introns. However, alignment of the putative promoters of these *PgPLs* (*PgPLD-alpha1-1*, *PgPLD-alpha1-5*, *PgPLD-delta1-7a*, *PgPLA1-II-1a*, and *PgPLD-delta1-2a)* with their corresponding homologous sequences revealed a multitude of variations in the form of single-nucleotide polymorphisms (SNPs), insertions and deletions (InDels). Notably, the putative promoter sequences of *PgPLD-alpha1-1* and *PgPLD-alpha1-5* exhibited conservation within the initial 500 bases (-1 to -500), with discrepancies observed beyond the − 500-nucleotide mark. Specifically, *PgPLD-alpha1-1* displayed SNPs at positions − 538, -628, -619, -637, -644, -647, -650, -652, -815, and − 877 along with deletions spanning from positions − 620 to -628, -728 to -735, and − 767 to -772. These alterations led to the inclusion of ROOTMOTIFTAPOX1, TATABOX4, and CAATBOX1 boxes in the *PLD-alpha1-1* promoters of genotypes PI583800, PI587025, PI526529, and PI521612 (Fig. 6a). Furthermore, the presence of DOFCOREZM, a key binding site for seed-specific Dof proteins characterized by a single zinc finger was solely detected in the *PLD-alpha1-1* promoter of genotype PI583800 but was absent in other genotypes. This finding aligns with its specific expression pattern in grains. Additionally, PI527388 and PI537069 showcased multiple copies of an enhancer element termed MARTBOX (Matrix Attachment Region for enhanced transcriptional activity).

SNPs were also identified within the putative promoter region of *PgPLD-alpha1-5* across six genotypes. These variations are specifically positioned at -454, -543, -671, -713, -715, -790, -791, -857, -881, -910, -913, -953, -966, and − 973. In addition, a deletion spanning from positions − 720 to -721 and an insertion at position − 824 are also observed. These modifications led to the incorporation of two additional copies of CACTFTPPCA1 and one copy each of DRE2COREZMRAB17, HEXAMERATH4, CGACGOSAMY3, GT1GMSCAM4, OSE2ROOTNODULE, ROOTMOTIFTAPOX1, NODCON2GM, CTRMCAMV35S into the *PgPLD-alpha1-5* sequence in the PI583800, PI521612 and PI526529 genotypes (Fig. 6b). While CTRMCAMV35S is an activator, both HEXAMERATH4 and GT1GMSCAM4 are induced by pathogen and hormonal signals.

Conversely, a major portion of the *PgPLD-delta1-7a* putative promoter sequence showed conservation across genotypes, with variations detected only at positions − 39 (C to A), -299 (A to G), -301 (A to G), -484 (C to T), and an additional base (T) inserted at position − 631. The conversion from C to A at position − 39 in PI583800 and PI526529 led to the inclusion of POLASIG3, while changes from A to G at positions − 299 and − 301 resulted in the deletion of INRNTPSADB in these genotypes. Furthermore, a change from C to T at position − 484 in PI526529 facilitated MYBCOREATCYCB1 addition (Fig. 6c). Finally, the introduction of base T at position − 631 was considered a synonymous mutation in both PI583800 and PI526529 genotypes. Likewise, the promoters of *PgPLA1-II-1a* exhibited a high degree of conservation among the six genotypes except for a single variation (G to A) observed at position − 919 within the PMH1G03645 promoter of the PI527388 genotype. This alteration introduced a POLASIG2 element known for its presence in alpha-amylase genes (Fig. 6d).

The upstream sequences of *PgPLD-delta1-2a* displayed a considerable array of variations. Notably, the alterations at positions − 256, -257, -258, -304, -371, -372, -373, -464, -465, -556, -679, and − 742 were found to be synonymous. Conversely, the mutations at -35, -95, -144, -199, -203, -263, -759, -783, -784, -820, -829, and − 906, along with deletions of TCACCA (-147) and AT (-651 and − 652) were considered non-synonymous. Importantly, the deletion of AT from P1583800 and P1587025 genotypes at -651 and − 652 positions resulted in the introduction of MYBPZM (a binding site for myeloblastosis-related transcription factors) and SEF4MOTIFGM7S (the Soybean Embryo Factor 4 binding site); these binding sites were not found in other genotypes (Fig. 6e). While MYB elements are known to play pivotal roles in organ morphogenesis, secondary metabolism, and response to external stimuli [48], SEF4MOTIFGM7S is responsible for regulating the expression of genes encoding beta-conglycinin seed storage proteins [49].

## Discussion

Pearl millet occupies a significant role within the realm of sustainable agriculture. This resilient crop serves as a crucial instrument in addressing poverty and ensuring food security in regions besieged by challenging climates, particularly arid and semi-arid areas worldwide [2]. A noteworthy attribute of pearl millet lies in its exceptional nutritional composition, rendering it a substantial source of nourishment for human diets in locales where dietary options are scarce [2]. The grains of pearl millet boast a wealth of essential nutrients, encompassing proteins, and fibers, as well as an array of vitamins and minerals. This nutritional richness positions it as a key staple food, especially for communities heavily reliant on it for sustenance. Despite its nutritional prowess, the shelf life of pearl millet flour is relatively short due to its vulnerability to hydrolytic and oxidative rancidity [17, 21]. Enzymes such as phospholipases and lipoxygenases catalyze the hydrolysis of storage lipids into free fatty acids (FFAs), instigating a swift deterioration in the quality of the pearl millet flour during storage [18].

Phospholipases stand out among enzymes for their robust characteristics and heightened efficacy under varied conditions. These enzymes serve a dual role by aiding in seed germination and contributing to the degradation of pearl millet flour through hydrolytic rancidity [50–52]. During the milling of pearl millet grains, endogenous phospholipases catalyze the hydrolysis of fatty acid ester bonds in triacylglycerol (TAG), leading to the release of FFAs and glycerol. Subsequently, these FFAs are oxidized by lipoxygenases, generating conjugated hydroperoxides that are decomposed by peroxidases into secondary oxidation products responsible for distinct undesirable off-flavours [17, 53]. Given the pivotal role played by phospholipases in initiating the rancidity pathway in pearl millet flour, this study is specifically focused on investigating the genes encoding phospholipases in pearl millet.

The utilization of comprehensive and high-quality genome sequences and database resources has enabled us to delve into the phospholipase gene family of pearl millet. Through a careful examination of MilletDB, a total of 44 genes belonging to the phospholipase gene family have been identified. These genes are dispersed across the seven chromosomes constituting the genome, with chromosome 1 exhibiting the highest gene count. We also presented a detailed expression analysis of phospholipase genes in pearl millet encompassing key growth stages and the identification of *cis*-regulatory elements from their putative promoter sequences. It appears that phospholipase genes are subject to developmental regulation given their widespread expression within all six tissues of two pearl millet genotypes. This is in harmony with the previous findings which also showed the differential transcriptional regulation of *PL* genes during various growth and developmental stages in other plants [51, 54–56]. Furthermore, we have identified genes with overlapping expression patterns across multiple tissues, as well as those specifically expressed in grains. We propose that targeting such grain-specific genes holds significant promise for enhancing various grain quality parameters of pearl millet including addressing rancidity issues through genetic or genomic engineering approaches.

The number of phospholipases identified in pearl millet exceeds the count in rice, which stands at 39 (RGAP-DB [57]). Additionally, pearl millet exhibits 21 PLDs as opposed to 16 in rice; furthermore, the number of PLA1 and PLA2 in pearl millet is also higher at 14 and 5 respectively, compared to rice’s 9 and 4. In pearl millet, the presence of PLD-alpha1 members is also notably higher, with a total of three (PgPLD-alpha1-1, PgPLD-alpha1-5, and PgPLD-alpha1-7) in contrast to rice which has only one (OsPLD-alpha1, *Os01t0172400*). It is also important to note that rice also undergoes rancidity in bran mediated by OsPLD-alpha1 [58]. The protein alignment analysis of OsPLD-alpha1 with PgPLD isoforms showed the highest resemblance with PgPLD-alpha1-1 and PgPLD-alpha1-5 proteins. Despite rice having a significantly larger number of total loci at 55,869 [57] compared to around 38,000 loci in pearl millet [59], the presence of phospholipases is more pronounced in pearl millet. The increased prevalence of PgPLD-alpha1 isoforms, PgPLDs, and overall PgPLs underscores the significant regulatory role played by phospholipases in flour rancidity issues of pearl millet, a phenomenon that is notably higher in pearl millet compared to rice and other agricultural produce. Moreover, the grain specificity and comparatively high expression of PgPLD-alpha1-1 and PgPLD-alpha1-5 which are homologues of OsPLD-alpha1 were noted in the high rancid than in the low rancid pearl millet genotype. Further elucidation and characterization of these identified genes hold promise for shedding light on their contributions to grain production - a pivotal trait influencing yield outcomes in pearl millet cultivation.

The categorization of PLDs into alpha, beta, delta, p1 and zeta isoforms is broadly based on their specific requirements for divalent cations, free fatty acids and the substrate vesicle composition [60]. The involvement of PLDs in diverse house-keeping activities such as vesicular trafficking, lipid remodelling, signal transduction, cytoskeletal rearrangement, membrane deterioration, cooking quality along stress responsiveness underscores their crucial roles in plant growth and adaptation to environmental cues [28, 30, 61–68]. Notably, PLD-alpha which is the most conventional and prevalent isoform operates independently of PIP2 and exhibits high efficacy at millimolar calcium levels [69]. In contrast, PLD-beta necessitates a poly-phosphoinositide cofactor for activation and shows optimal activity at micromolar calcium concentrations [70, 71]. PLD-gamma which has not been identified in pearl millet displays reduced dependence on poly-phosphoinositide and calcium binding for functionality [70]. Unlike other PLDs, PLD-delta is activated by oleic acid [72–74]. Previous studies strongly suggest an important role for this protein in abiotic stress tolerance and plant innate immunity [75–78]. The presence of four variants of PLD-delta isoforms in pearl millet might be associated with its high resilience to varying climatic conditions. Distinct from other PLDs, PLD-zeta functions independently of Ca^+ 2^, relying solely on PIP(2) for its enzymatic activity [60].

The PLC enzymes demonstrate specificity towards three substrates, namely PIP2 (Phosphatidylinositol 4,5-bisphosphate), PIP (phosphatidylinositol phosphate), and PI (phosphatidylinositol) [27]. The breakdown of PIP2 results in the production of diacylglycerol (DAG) and IP3 (Inositol trisphosphate). DAG triggers the activation of Ca^+ 2^-dependent protein kinase C (PKC), which subsequently phosphorylates downstream signalling molecules like AKT (serine/threonine kinase). This cascade initiates various cellular processes such as the regulation of cell proliferation, cell polarity, and spatial distribution of signals [79, 80]. On the other hand, IP3 binds to its corresponding receptors on the endoplasmic reticulum, inducing the release of intracellular calcium which generates the characteristic calcium spike that acts as a signal for cell activation [81]. The involvement of *OsPLC1*, a member of the rice PI-PLC family, extends beyond its function in responding to abiotic stresses. It also plays a significant role in controlling grain size, as demonstrated by the contrasting grain sizes observed in *osplc1* mutants and lines with elevated expression levels of *OsPLC1* [82, 83]. Among the different types of PLCs (β, γ, δ, ε, η, and ζ), only delta isoforms were identified in pearl millet based on motif analysis. The delta isoforms of PLC are highly responsive to calcium and have been associated with diverse signaling responses triggered by environmental stresses in various plant species [84–90]. Knocking out these delta isoforms often results in embryonic lethality [91]. The prevalence of PLC-delta isoforms in pearl millet may contribute significantly to its ability to thrive under arid conditions.

PLCs and PLDs are categorized within the phosphodiesterase group, whereas PLAs, specifically PLA1, PLA2, and patatin-like PLAs are affiliated with the acyl-hydrolase family and possess the α/β hydrolase domain [92]. PLA1 is responsible for cleaving the sn-1 acyl chain, while PLA2 hydrolyzes the sn-2 acyl chain and patatin-like PLAs exhibit activity towards both positions [22, 25, 92]. Other members of the lipase family, including monoacylglycerol lipase, triacylglycerol lipase, and lipoprotein lipase, are also classified under PLA since they can cleave both the sn-1 and sn-2 acyl chains [92]. In our study, we have identified 19 members of PgPLAs, including 14 PgPLA1 and 5 PgPLA2; however, no patatin-like PgPLAs were detected. Among these members, two PgPLA1-DAD1 isoforms were identified. DAD1 is a sn1-specific acyl-hydrolase known for its significant roles in regulating cellular processes such as seed germination, seedling establishment, and tissue growth [93]. Consistent with our findings, PLA1 transcripts have been reported to be present in almost all plant organs, with different isoforms displaying varying degrees of tissue specificity [92]. Furthermore, all *PgPLA1* members feature multiple copies of stress-responsive *cis*-elements, suggesting their involvement in spatiotemporal and stress-related processes. PLA1 enzymes play a crucial role in various physiological processes in plants, including the response to jasmonic acid, defence against UV light-induced damage, signaling pathways, the initiation of senescence, seedling establishment, seed viability and longevity maintenance, as well as cell development, tissue growth, and stress responses [94–99]. In line with previous studies emphasizing the involvement of PLA1s in jasmonic acid responsiveness, our investigation has identified MeJa-responsive *cis*-elements in ten out of the 14 *PgPLA1* putative promoters.

On the other hand, PLA2s in plants represent a group of secreted phospholipases characterized by a relatively smaller and less complex array of proteins compared to PLA1s [100, 101]. In Arabidopsis and rice, only four PLA2 isoforms have been identified [57, 102], whereas our analysis has unveiled five *PgPLA2* isoforms in pearl millet. Analogous to animal and other plant PLA2s, *PgPLA2* members also demonstrate low molecular masses ranging from 15 to 17 kDa. Additionally, akin to PLA1 enzymes, PLA2 transcripts are expressed across various plant tissues. Notably, transcripts of *PgPLA2-3b* were discerned in all six tissues examined from both genotypes of pearl millet. Crucially, PLA2 enzymes are also implicated in plant growth and defence mechanisms against stresses [24, 25, 103, 104].

Plant genetic resources encompass the entirety of the hereditary material present in a particular crop species, including its wild counterparts. This genetic material consists of all alleles of various genes and promoters, resulting from genetic recombination and mutations. Consequently, it leads to a wide array of variations in epigenetic profiles, and protein structure and function, ultimately influencing the diverse physiological and morphological traits observed in plants [105]. Promoter sequences play a crucial role in determining genetic diversity, influencing the variability in gene expression, and providing resilience to genetic variants [106]. In this study, we explored the potential variations in putative promoter sequences of five grain-specific genes across six different pearl millet genotypes. Our analysis revealed a multitude of SNPs and deletion mutations leading to the formation of distinct transcription factor binding sites. These variations, in conjugation with others, are likely to contribute to the differential expression patterns of phospholipases under varying environmental conditions and could have potentially aided in the development of resilient pearl millet genotypes.

## Conclusions

This study employed a genome-wide computational analysis to characterize the phospholipase gene family in pearl millet, marking it the first comprehensive report on phospholipases in pearl millet. Investigation of expression profiles of 44 phospholipases across six different tissues of two contrasting pearl millet genotypes indicated the widespread distribution of their transcripts. In the high rancid genotype (ICMB-863), the highest number of transcripts were detected in grains, comprising 34 genes. Subsequently, the seed coat exhibited expression of 25 genes, followed by the endosperm with 23 genes, immature embryos with 21 genes, and glumes with 14 genes. Notably, only seven genes showed expression in the leaves. Conversely, in the low-rancid genotype (ICMB-95222), the highest number of phospholipases were found in grains, totalling 31 genes. A substantial number of genes were expressed in the leaves (28 genes), followed by the seed coat (19 genes), glumes (14 genes), endosperm (13 genes), and immature embryos (12 genes). Amongst the 30 phospholipase genes exhibiting grain-specific expression across both genotypes, *PgPLD-alpha1-1, PgPLD-alpha1-5, PgPLD-delta1-7a, PgPLA1-II-1a*, and *PgPLD-delta1-2a* were identified. This underscores their potential utility in enhancing grain quality traits and tackling the persistent problem of rancidity in pearl millet.

## Materials and methods

### Nucleotide sequence retrieval, nomenclature and synteny of phospholipase genes

To ascertain the total count of members within the phospholipase gene family of pearl millet, an exploration was initiated by employing the keyword ‘phospholipase’ in the search engine tool accessible under the ‘Gene’ option of the Novogene Millet database [107]. This inquiry yielded over a thousand results in the Novogene MilletDB.^1^ Genotype PI583800 from the Indian sub-continent was considered as a reference genotype, leading to the identification of a total of 65 phospholipases (PLs) including both specific and pseudo members. The protein sequences of these 65 potential candidates were scrutinized in the NCBI conserved domains database (NCBI-CDD^2^) to determine the presence of conserved domains. The phospholipases of pearl millet were also compared with those present in rice. To identify the rice phospholipases, a search query was performed using the keyword ‘phospholipase’ in the putative function search option of the Rice Genome Annotation Project Data Base (RGAP-DB 7).^3^

In plants, four primary categories of phospholipases are established based on their substrate specificity and catalytic activities [22]. These include phospholipase A1 (PLA1), phospholipase A2 (PLA2), phospholipase C (PLC), and phospholipase D (PLD). In the nomenclature process, a systematic approach was adopted whereby these enzymes were denoted starting with the abbreviation *Pg* to signify their association with the genus *Pennisetum glaucum*, followed by their respective class designation (*PgPLA1, PgPLA2, PgPLC*, or *PgPLD*). Subsequent differentiation was achieved through the allocation of specific isoforms such as alpha, beta, delta, or zeta. Furthermore, in instances where multiple isoforms existed, they were further delineated based on their distinct chromosomal loci.

The syntenic analysis of pearl millet phospholipases was conducted with those of other species by accessing genomic data from the Ensembl Plants Database^4^. for Arabidopsis (*Arabidopsis thaliana*), rice (*Oryza sativa*), sorghum (*Sorghum bicolor*), finger millet (*Eleusine coracana*), and foxtail millet (*Setaria italica*). The genome sequence of pearl millet was obtained from the International Pearl Millet Genome Sequencing Consortium^5^ (IPMGSC) database. The collinearity among phospholipase gene pairs was established using the TB-tools^6^.

### Chromosomal distribution of phospholipase genes

The chromosomal position of *PLs* was determined by submitting the locus numbers of 44 candidates to the gene mapping tool of MilletDB, specifically selecting the PI583800 genotype option. Subsequently, the location of each candidate at its corresponding locus on the chromosome was manually identified based on the output obtained. The chromosomal map of phospholipases in rice was generated by submitting the phospholipase locus IDs obtained from RGAP-DB to OryGenesDB^7^. The resulting output produced a map that illustrates the genomic distribution of phospholipases in rice.

### Phospholipase gene structures

To gain insights into the phospholipase gene structure such as the gene size, the number of introns and exons, as well as the GC content, the cDNA and gene sequences were extracted from the Novogene MilletDB and the genome browser, respectively.

### Protein Properties, phylogeny, secondary structure prediction and regulatory network analyses

The protein sequences of 44 PgPLs were analyzed using ExPASy ProtParam^8^, an online tool known for its ability to forecast various protein properties such as size, molecular weight, and isoelectric point (*p*I). The alignment of the amino acid sequences of these proteins was accomplished through ClustalW^9^. An unrooted phylogenetic tree was constructed to discern resemblances within the PL protein family of pearl millet genotype PI583800. MEME Suite 5.5.5^10^ was employed to identify enriched amino acid sequences within each protein clade. Additionally, to explore the evolutionary links between rice and pearl millet phospholipases, as well as to identify their similarities and variations, the 39 phospholipase protein sequences from rice sourced from RGAP-DB were employed in ClustalW to construct an unrooted phylogenetic tree.

The overall hydrophobicity of the PgPLs was assessed by computing the GRAVY (Grand average of hydropathicity) indices using ExPASy ProtParam. Typically, the GRAVY indices of all PLs fell below zero with negative values indicating a hydrophilic nature [108]. To predict the three-dimensional secondary structure of the 44 PL proteins, we employed the Phyre2^11^ program (Protein Homology/AnalogY Recognition Engine v2) [109]. Protein sequences were formatted to FASTA and analyzed for characteristics such as α-helices, β-strands, and distorted portions. To elucidate the metal and non-metal ligands interacting with PLs, the individual sequence was initially processed through the Protein Data Bank^1^^12^ (RCSB-PDB) to procure the PDB code, which was subsequently analyzed via SuMo version 5^13^. The computational modelling of protein-protein interactions among PLs was executed using the STRING v11^14^ platform, known for predicting networks based on functional relationships [110]. A high confidence threshold score of 0.7 which was widely reported [111] was also applied in this investigation.

### Plant material and growth conditions

The seeds of two contrasting pearl millet genotypes for rancidity, ICMB-863, designated as the high rancid genotype and ICMB-95222, the low rancid genotype [112, 113], were procured from the pearl millet crop breeding group of the International Crops Research Institute for the Semi-Arid Tropics (ICRISAT), located in India. A portion of these seeds was utilized for RNA isolation, while others were directly sown in black alluvial soil and cultivated within a controlled environment in the glasshouse under a 16-hour light/8-hour dark photoperiod at a temperature of 30 ± 2 °C with a relative humidity of 70 ± 5%. To investigate the expression patterns of 44 phospholipase genes in pearl millet, tissue samples were collected from six distinct plant parts, including young leaves, immature embryos, endosperm, glumes, seed coats, and grains. Young leaves were collected at the 35-day growth stage of the plant, whereas seed coat, endosperm, and glumes were collected when the plants reached an age of 85 days; immature embryos were obtained from plants aged 90 days. The collection of tissue samples was carried out in biological triplicates from three separate plants that were all subjected to the same growth conditions.

### RNA isolation, cDNA synthesis, and quantitative-PCR (qRT-PCR)

Total RNA was extracted from six different tissues of two contrasting genotypes of pearl millet (ICMB-863 and ICMB-95222) using TriReagent (Takara Bio, UK) according to the manufacturer’s guidelines. The quality of the isolated RNA was checked on a 2% agarose gel in TAE (Tris-acetate-EDTA) buffer and quantified using a Qubit-4 fluorometer (Thermo Fischer, US). Subsequently, 2 µg of total RNA was employed for the synthesis of the first strand of cDNA using reverse transcriptase (Takara Bio, UK). The cDNA was subjected to a 1:5 dilution, and 1 µl of this dilution was used in a qRT-PCR reaction mix. The primers specific to each phospholipase gene, whose sequences were obtained from the MilletDB, were designed using the online primer-3^15^ tool. A concentration of 10 µM for each primer set was used per reaction. To ensure the specificity of primers, they were checked in NCBI-BLAST^16^ against the *Cenchrus americanus* (taxid: 4543) genome. The qRT-PCR reaction conditions included an initial denaturation at 95 °C for 2 min, followed by 30 cycles consisting of secondary denaturation at 95 °C for 30 s, an appropriate annealing temperature for 25 s, and extension at 72 °C for another 25 s. A melting curve step was integrated post-reaction to assess the amplification specificity of each gene. Each qRT-PCR reaction underwent two biological and three technical replicates. The relative transcript levels of phospholipases were normalized using the internal reference gene *eIF4*, with mean fold changes depicted as bar diagrams generated via SigmaPlot v11 software. Statistical significance was determined through a one-way ANOVA at a significance level of *P* < 0.05.

### *In silico* identification of *cis-*regulatory motifs

To identify the various *cis*-regulatory elements, approximately 1 kb upstream nucleotide sequence of each *PL* gene was retrieved from the Novogene Millet genome browser. This sequence was then submitted to the PlantCARE^17^ database, to determine the location and number of repeats of *cis*-regulatory elements in *PL* genes.

To investigate the genetic variations in the promoter sequences of five grain-specific genes (*PgPLD-beta1-5a*, *PgPLA1-II-6d, PgPLD-beta1-1b, PgPLA2-2*, and *PgPLD-delta1-2b*) among different millet genotypes, the upstream putative promoter sequences of these genes from Indian genotype (PI583800) were compared with corresponding homologous sequences of five other millet genotypes. Variations and resulting alterations in *cis-*acting elements were identified through this comparative analysis. This study encompassed three wild, two cultivated, and one landrace genotypes of pearl millet (PI583800, PI587025, PI527388, PI537069, PI521612, and PI526529). Among these genotypes, PI583800 and PI527388 are cultivated variants from India and Algeria, respectively. The wild genotypes included PI537069 from Niger, PI521612 from Kenya, and PI526529 from Zimbabwe. Additionally, a landrace genotype from Saudi Arabia was represented by PI587025. To accomplish this investigation, the 1 kb upstream sequence starting from the initiation codon of each homolog was extracted using the pearl millet genome browser and aligned using the ClustalW online tool. Subsequently, the *cis*-element analysis was conducted by inputting the promoter sequences of the six genotypes into the PLACE^18^ database individually.

### Electronic supplementary material

Below is the link to the electronic supplementary material.

Supplementary Material 1

## Data Availability

Data supporting the findings of this work are available within the paper and its supplementary files. The DNA and protein sequencing data that were used for the current study are available in the Novogene MilletDB (http://milletdb.novogene.com/home/).

## Footnotes

1 http://milletdb.novogene.com/search/.

2 https://www.ncbi.nlm.nih.gov/Structure/cdd/wrpsb.cgi.

3 http://rice.uga.edu/.

4 https://plants.ensembl.org/index.html.

5 https://cegsb.icrisat.org/ipmgsc/index.html.

6 https://bio.tools/tbtools.

7 https://orygenesdb.cirad.fr/.

8 https://web.expasy.org/protparam/.

9 https://www.genome.jp/tools-bin/clustalw.

10 https://meme-suite.org/meme/tools/meme.

11 http://www.sbg.bio.ic.ac.uk/phyre2/html/page.cgi? id=index.

12 https://www.rcsb.org/.

13 http://sumo-pbil.ibcp.fr/cgi-bin/sumo-welcome.

14 https://string-db.org/.

15 https://primer3.ut.ee/.

16 https://blast.ncbi.nlm.nih.gov/Blast.cgi.

17 https://bioinformatics.psb.ugent.be/webtools/plantcare/html/.

18 https://www.dna.affrc.go.jp/PLACE/?action=newplace.
