# Supplementary material for: Exploration of the pearl millet phospholipase gene family to identify potential candidates for grain quality traits
Source: BMC Genomics. 2024 Jun 10;25:581. doi: 10.1186/s12864-024-10504-x (PMC11165789; doi:10.1186/s12864-024-10504-x)
Supplement: Supplementary file 1 — Supplementary Material 1 [file 12864_2024_10504_MOESM1_ESM.docx]

**Supplementary Fig. 1. Chromosomal distribution of rice phospholipases**

**
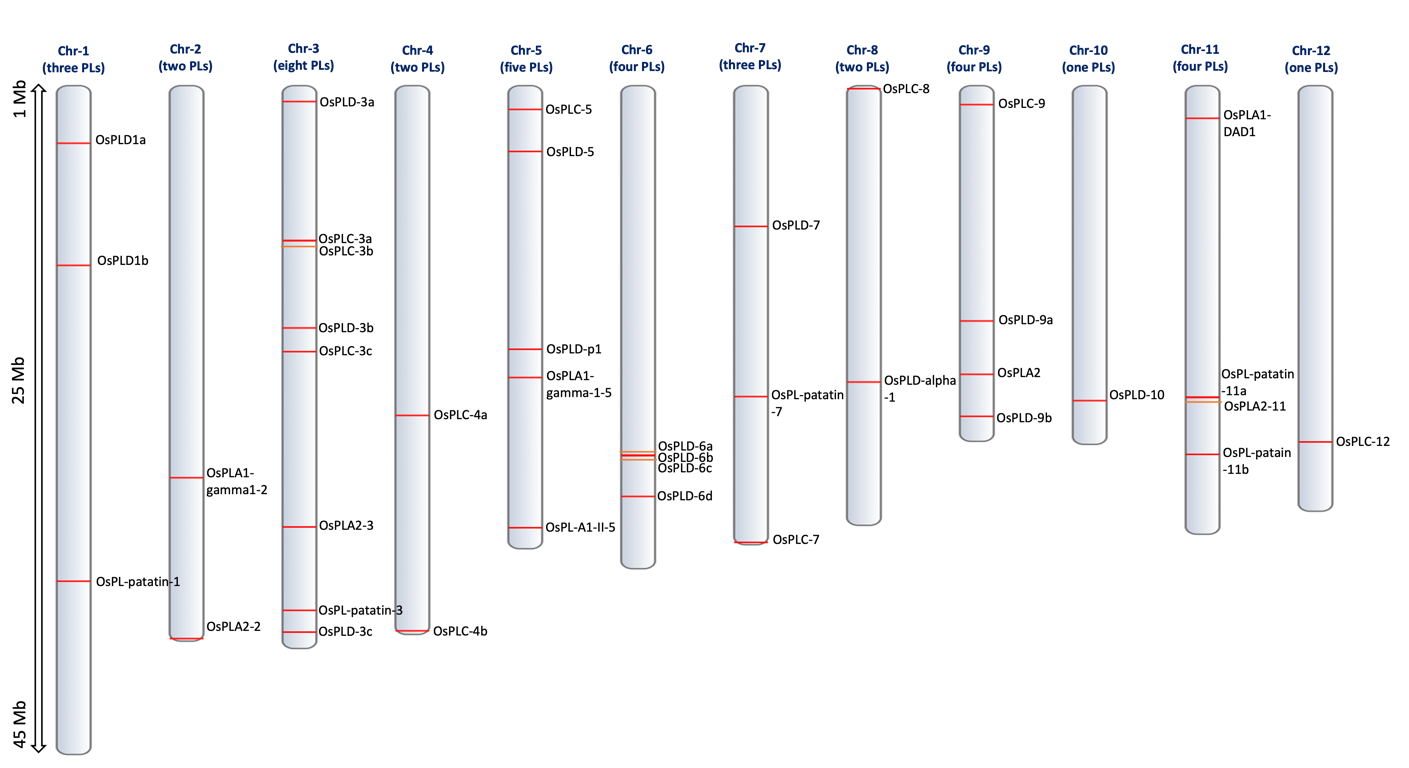
**

**SFig.1.** Distribution of 39 phospholipases across 12 rice chromosomes. The position of each phospholipase gene is highlighted by red horizontal bars on the chromosome, with the chromosome number provided at the top along with the total number of phospholipases in brackets. A scale measured in megabase (Mb) is included on the left side of the map.

**Supplementary Fig. 2. An integrated phylogenetic analysis of rice (OsPLs) and pearl millet (PgPLs) phospholipases**

**
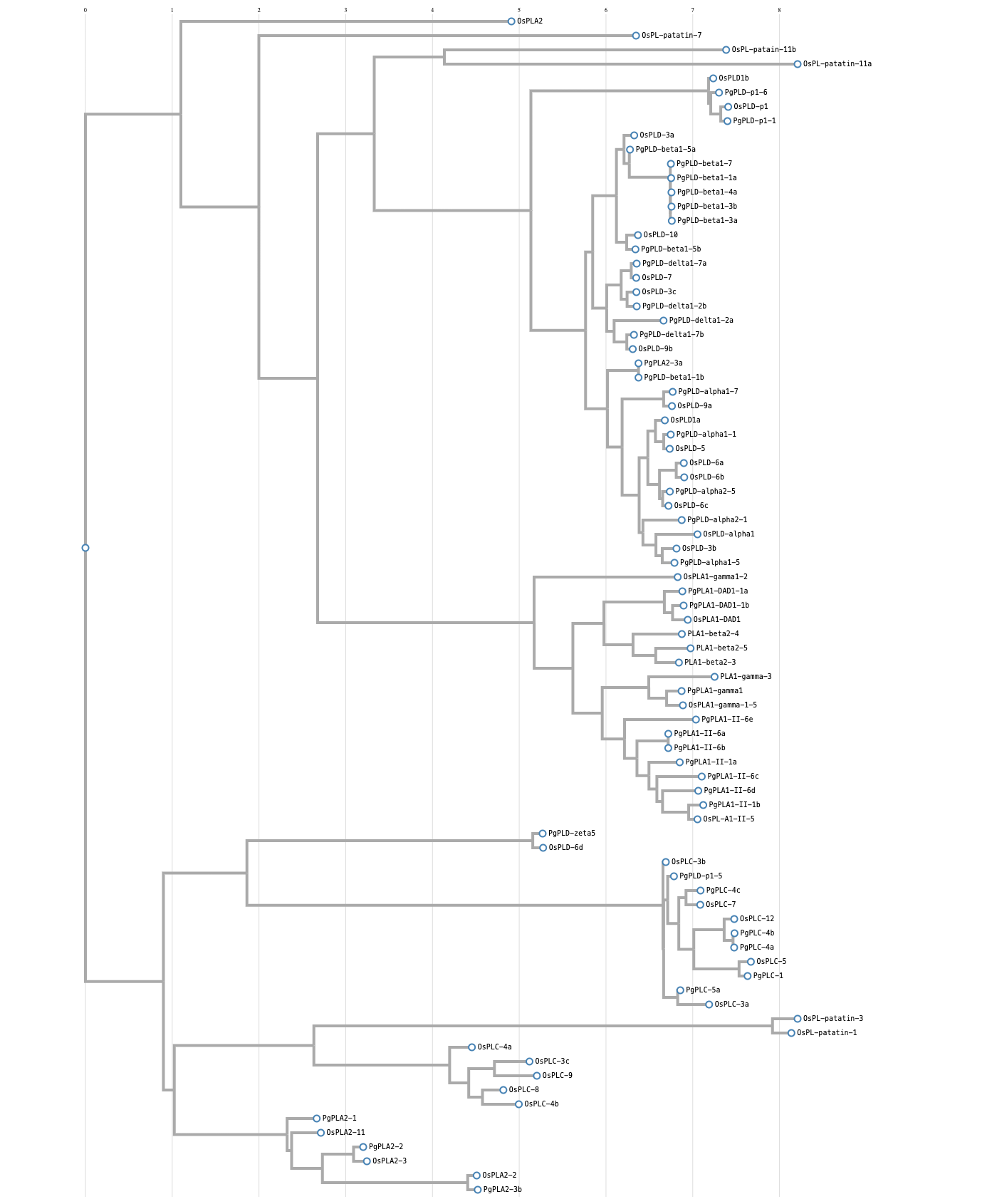
**

**SFig. 2.** The phospholipase protein sequences from rice and pearl millet were grouped based on their specific isoforms. Notably, OsPLD-1b and PgPLD-p1-6 shared similarities as they clustered together in a common clade. Similarly, OsPLD-3 and Pg-PLD-beta1-5a were grouped under one clade, together with OsPLD10 and Pg-PLD-beta1-5b. Furthermore, OsPLD7 and PgPLD-beta1-5b demonstrated resemblance by being placed in the same clade. Additionally, OsPLD-3b and PgPLD-delta1-2b were identified to belong to a shared clade, while OsPLD-9b and PgPLD-delta1-7b were positioned in the same group. Moreover, both OsPLA1-DAD1 and PgPLA1-DAD1 were observed in the same cluster. Similarly, OsPLA1-gamma1-5 and PgPLA1-gamma1 were housed within the same clade, as were OsPLA1-II-5 and PgPLA1-II-1b. PgPLD-zeta5 and OsPLD-6d fell into a unified clade; similarly, OsPLC-7 was associated with PgPLC4c, while both OsPLC-12 and PgPLC4a and 4b resided in the same group. Also, both OsPLA2-2 and PgPLA2-3b appeared under one clade, just like OsPLA2-11 and PgPLA2-I which were grouped together as well.

**Supplementary Fig. 3.** In silico association networks of phospholipases of pearl millet.

**
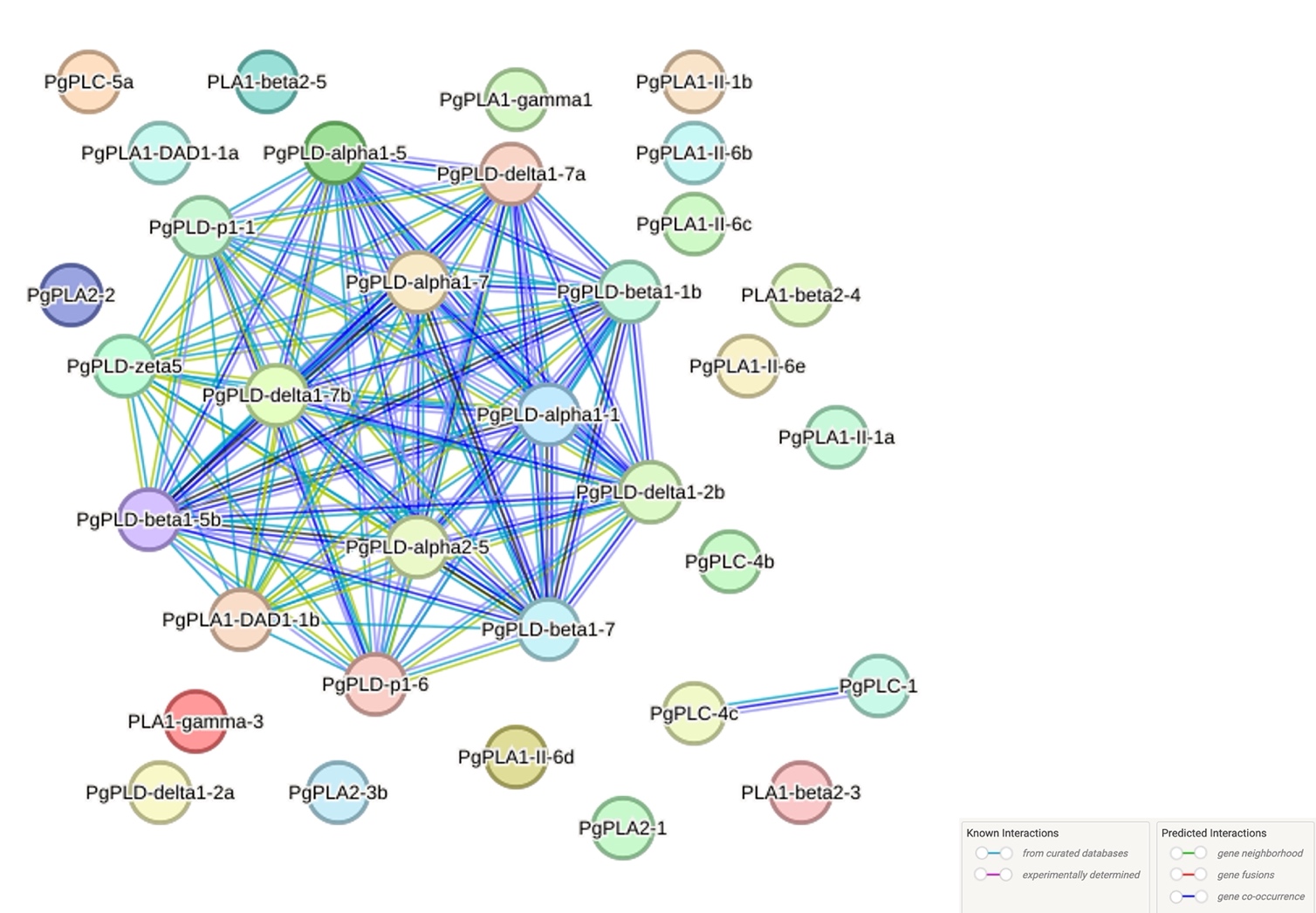
**

**SFig. 3.** The network analysis of 44 phospholipases was performed in the STRING database, of which approximately 10 nodes were not part of this interaction. The protein nodes that were part of the network were MCL-clustered (Markov Cluster algorithm) with an inflation parameter of three. The type of node interaction was depicted with different colors, which were provided at the bottom left corner.

**Supplementary Fig. 4.** Pictorial representation of tissues that were used for sample collection

**
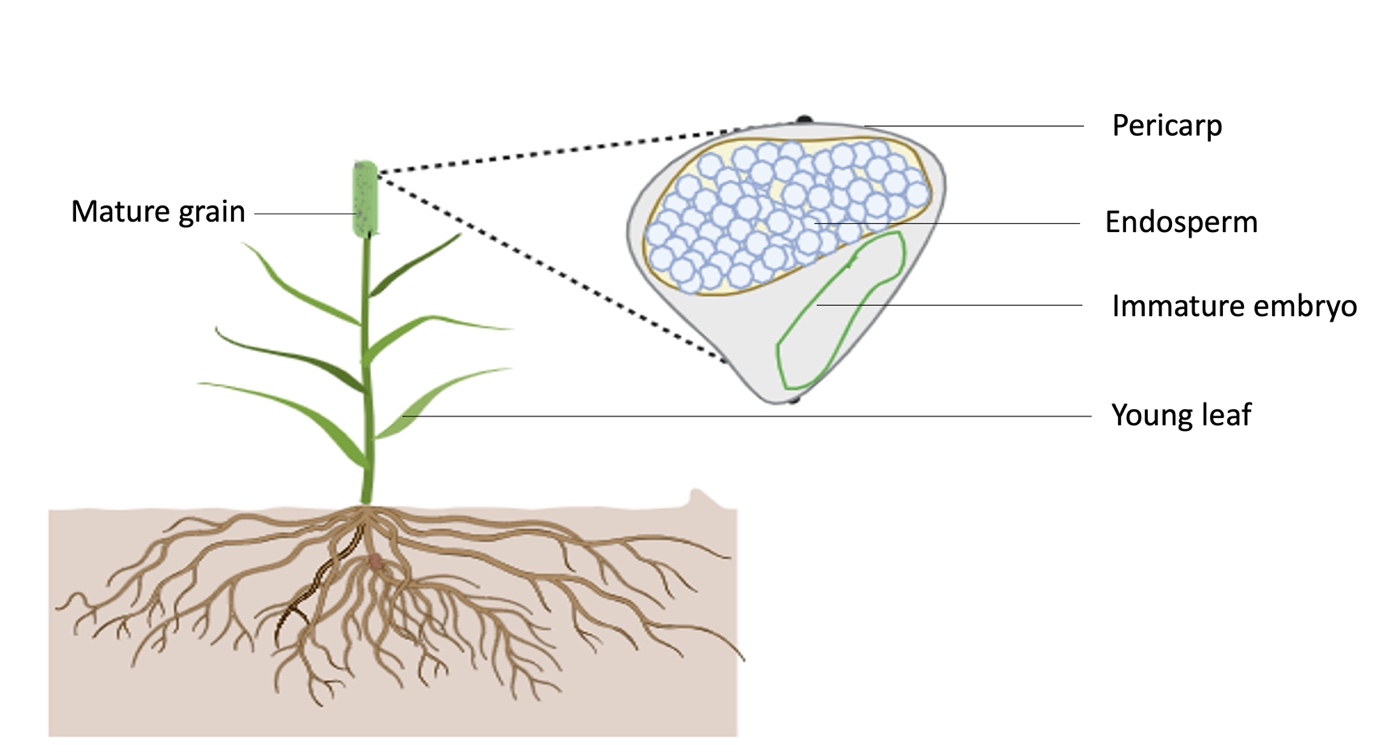
**

**Supplementary Fig. 5.** Venn diagrams representing overlap in expression of phospholipases between six tissues of two genotypes

**
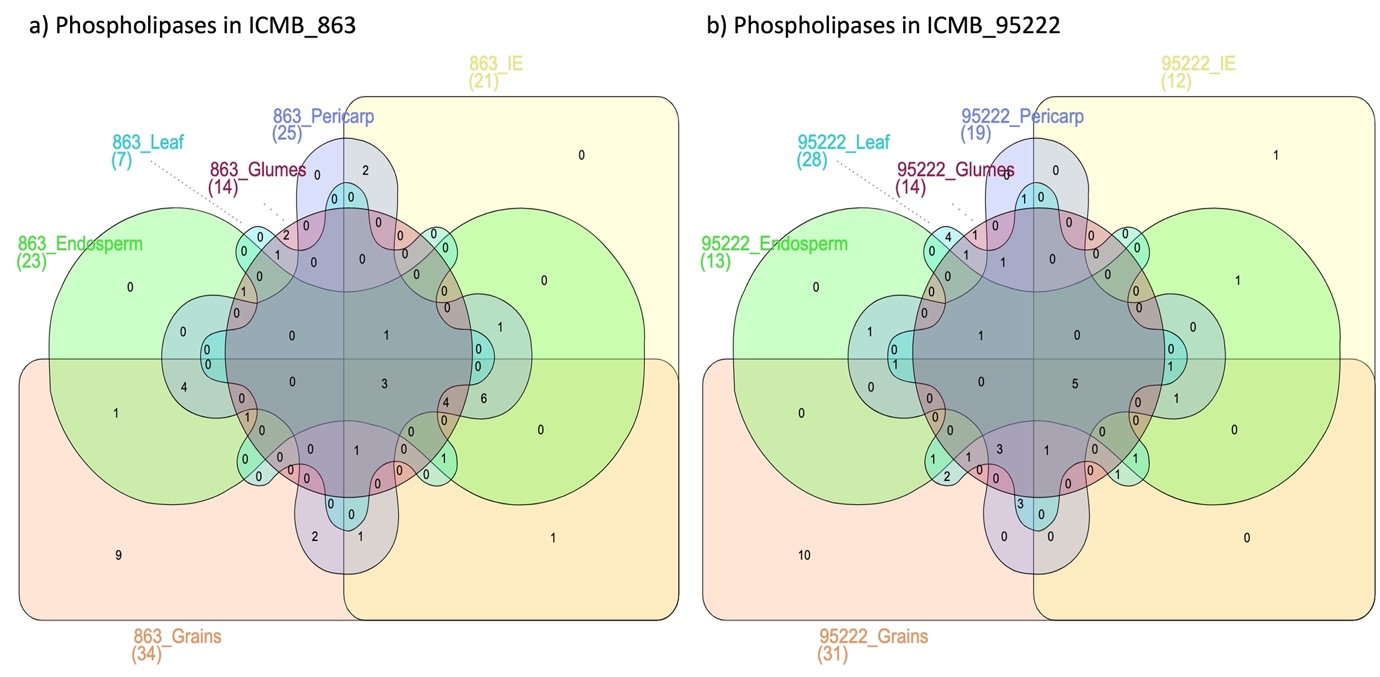
**

**Supplementary Fig. 6. A flowchart representing the different *in silico* analyses performed on the phospholipase gene family of pearl millet**

**
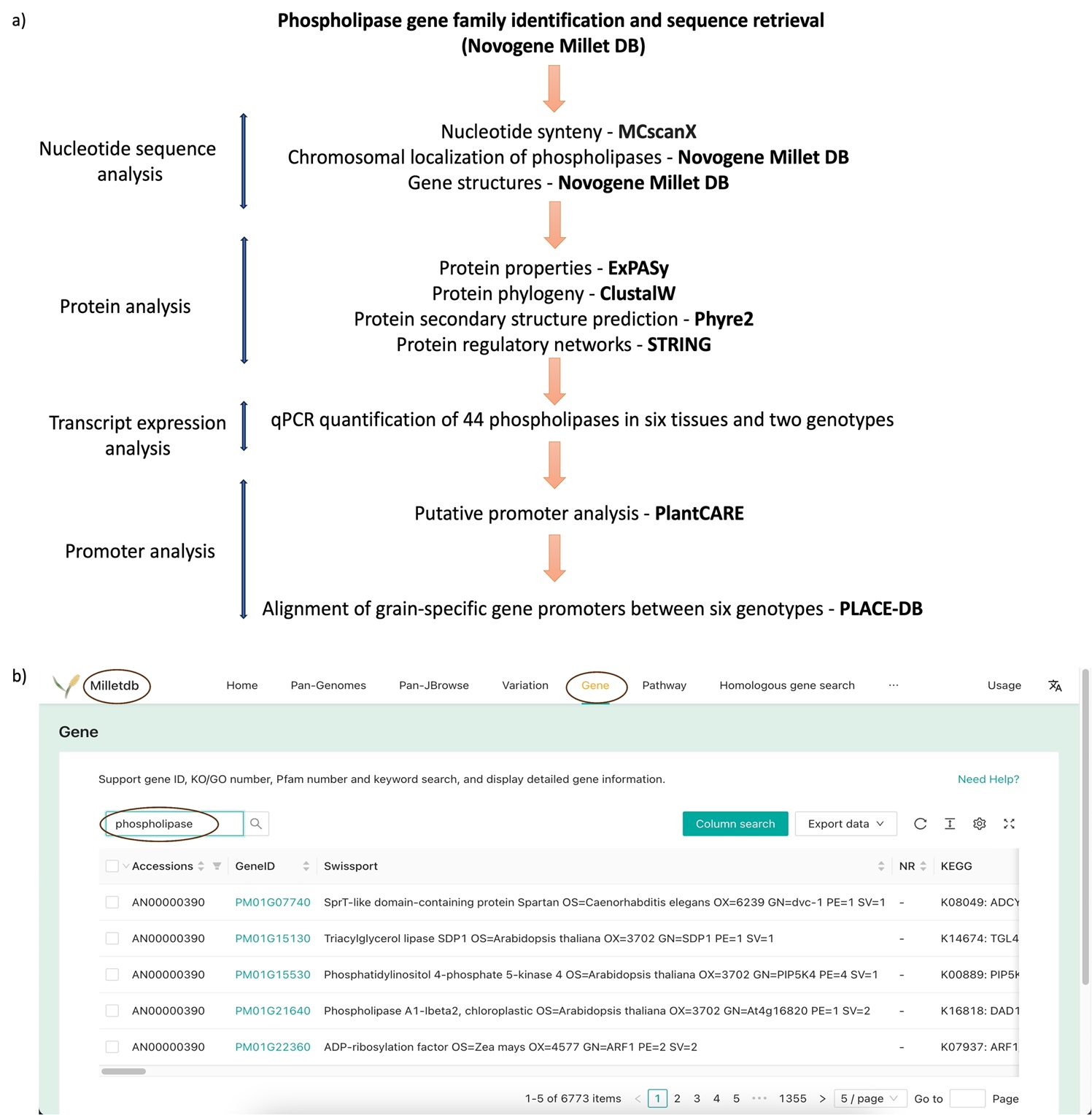
**

1. A flowchart illustrating the different computational analyses performed on the phospholipase gene family of pearl millet.
2. A Novogene Millet DB screenshot displays the gene search engine, which produced approximately 6,773 results across 18 genomes, including 11 pearl millet genotypes listed in the database when the keyword "phospholipase" was used. Among these results, 65 PLs from the PI583800 genotype were chosen for analysis. The protein sequences of these 65 candidates were examined in the NCBI conserved domains database (NCBI-CDD) to determine the presence or absence of conserved domains. As a result, a total of 44 phospholipases were identified and selected for further investigation.

**Supplementary Table 1. Details of pearl millet phospholipase genes**

**Supplementary Table 2: Primer sequences of phospholipase genes used for qPCR**

| **SNo** | **Gene ID** | **Forward primer** | **Reverse primer** |
| --- | --- | --- | --- |
| 1 | PMD5G02305 | CTTCCACCAGCCCAATTTCC | AAGTTGTCCAGAACGTCCCA |
| 2 | PMD7G03361 | CATGTCGTCAGCTTCATCGG | CAGGCGTGCTTGTAGTTGTT |
| 3 | PMD1G00695 | GTCGACGTCAAGAGGGATGG | CTGCCAACATTCACCAGCAC |
| 4 | PMD1G00694 | CGCTCAAGATCGTCAGCAAG | ATCGTCTTCCTCTGCCAGTG |
| 5 | PMD5G04890 | GATCACCCACCACCAGAAGA | GTGGAACTGTGTGTCGTAGC |
| 6 | PMD1G03427 | GATCTGCTCAGGAGGAAGTCG | GTAGCCTTCCATCTTGATGCC |
| 7 | PMD2G07986 | CCGTGTCATTCTCTACCAGGA | AAATGTCCTCCCAACACTTGC |
| 8 | PMD3G07688 | GATACTGCTTTGCCCACGATC | ACACACATGAACGCCTTCTTC |
| 9 | PMD5G01607 | GGCACAAAGACTTCAAGCAGA | GGCTTTCGTCTTCCTCATCAC |
| 10 | PMD7G04652 | GATCTGCTCAGGAGGAAGTCG | GTAGCCTTCCATCTTGATGCC |
| 11 | PMD7G05807 | GTGCATTGTGTGTTGTCTCCT | TTGTTTCCTGTGGCTTGAGTG |
| 12 | PMD7G02029 | CAGATGAACTCAAGGCCATGG | CTGAATCCGAGCTACCGTTTG |
| 13 | PMD1G04280 | ATAGTCCTGGGCACAAAGTCA | TAGCACGGTCCAGTTCATCTT |
| 14 | PMD1G04489 | AGGACGAGAACAAGGGCATG | CTATGATGGTGGTGTTGGTGC |
| 15 | PMD1G07144 | CGTACGAGAAGGCCATAGTCT | TGTAATTGGACGACGTGATGC |
| 16 | PMD1G08120 | GTGGTGAACTCGAACGACATC | CAACCTCACCGCATCATTCG |
| 17 | PMD1G08121 | GCTCGACAACTTCAAGTCCAG | GTGAAGAGCCTCCAGAACCC |
| 18 | PMD1G06220 | GGATTCAAGCTGGAGGTGGA | GCCCTTGACCATGAACTTGTT |
| 19 | PMD1G06219 | CTGGTGAACAAGGCGTACGA | CCTCGCAATCCATCAATCTCC |
| 20 | PMD3G08871 | TGAAATGCCACGAGAAGTTCA | GCCATGTCCATTCCTTGAGTC |
| 21 | PMD4G00298 | TCTGGAACCTCTACAAGACCG | ACCTCCTCGCCCTTGTATTG |
| 22 | PMD4G01357 | TGAAGGCGGATAGTGTGATGA | CTCCTGCACCTCTATCCTCAG |
| 23 | PMD4G01436 | CCAAGGACGACATCAACATCC | TCGAGCGTTATGATGACAGGA |
| 24 | PMD5G04439 | GATTTTGACCAAGAGAGCGCT | TCCAGACTTTTGCATGCACAA |
| 25 | PMD5G03093 | GTCTCCTCTCAACCCGAACTC | GAGGAGGCGGTCGAGGTTAG |
| 26 | PMD6G02138 | AGAAGGCCTCACAAGTGCTAT | GCTCCCCGATTCCCAAATTTT |
| 27 | PMD5G01606 | TCTAAAGGGGCGTATCATGCT | CCCCATGCAGCTTCTTCTTTT |
| 28 | PMD6G04193 | GGGCTTCTTGTCTGTGTACAC | ATCCAGCAGCCTCTTAATCTCA |
| 29 | PMD6G04201 | TTGGTTGATCGAGACATTGCC | TCCTTGCCTTTCACCATACCT |
| 30 | PMD6G04790 | TCGTGAATGGGAAGGTCGTC | ACGCCGTGAAAAGATTAGCTC |
| 31 | PMD5G00144 | AGGGTCAGCTAACATCAACCA | GTACCCGAAAATCTGTCCACG |
| 32 | PMD6G00496 | GCTAAGTGACATGGGCATCC | GCGCAGAGTACCTACAGAATC |
| 33 | PMD4G05665 | GATGACCAGCGACCCCTAC | CGACATTGACGACGAAGTGC |
| 34 | PMD1G01583 | GTTATGGCAAGTACTGCGGC | CAGGTAGTCGTTGTCGGTGG |
| 35 | PMD2G06735 | TCCAGGCAAAGAAGGACTACC | CTCGTCGATCATGCACTTGTT |
| 36 | PMD4G01333 | TAGAGGTGCAGGAGTACGACA | CCTGACCGACTTGTACCTGAC |
| 37 | PMD3G07336 | GCCGTACAAGAAGGAGCAGTA | CGTCGATGAGGTGCAGGTATG |
| 38 | PMD6G04197 | CGTCACGAGGTTCCTGTACG | GCGACGTATCCCATCCAGTT |
| 39 | PMD6G04200 | GCATCCGCCTACAACAAACC | GAGGCGGTGGAAGATGTCTC |
| 40 | PMD2G00129 | ACTTCATCGGCTCGTCGTAC | CACATGGGCATCACGACGTA |
| 41 | PMD3G04495 | ATGGAAGGCTACATGGGCAC | ATGACGGGGCACAGGATTTC |
| 42 | PMD5G02728 | GTGGGGTGGTCAGTTTTCCA | AAACAAGAAGCAACACCCGC |
| 43 | PMD3G06955 | GGAAGTACTGCGGGATCCTG | CCGCGTGTTCAGGTAGTCAT |
| 44 | PMD1G06693 | TCACGCACCACCAGAAGATC | TCCCAAACAGAGAGTGGCAC |

**Supplementary Table. 3. Detailed analysis of expression of phospholipases in different tissues of two genotypes of pearl millet**

**3a. Details of phospholipases with an overlap in expression in different tissues of ICMB_863 genotype**

| **Tissue type** | **No. of candidates expressed** | **List of candidates** |
| --- | --- | --- |
| 863_Endosperm, 863_Glumes, 863_Grains, 863_Immature Embryo, 863_Leaf, 863_Pericarp | 3 | PgPLD-zeta5, PgPLA2-3b, PgPLC-delta-4b |
| 863_Endosperm, 863_Glumes, 863_Grains, 863_Immature Embryo, 863_Pericarp | 4 | PgPLD-delta1-7b, PgPLD-beta1-5a,  PgPLD-p1-5, PgPLA1-II-6d |
| 863_Glumes, 863_Grains, 863_Immature Embryo, 863_Leaf, 863_Pericarp | 1 | PLA1-beta2-3 |
| 863_Endosperm, 863_Glumes, 863_Immature Embryo, 863_Leaf, 863_Pericarp | 1 | PgPLC-delta-4a |
| 863_Endosperm, 863_Grains, 863_Immature Embryo, 863_Pericarp | 6 | PgPLD-beta1-5b, PgPLD-beta1-3a, PgPLA2-2, PgPLD-p1-1, PgPLD-beta1-7, PgPLD-beta1-1a |
| 863_Endosperm, 863_Grains, 863_Immature Embryo, 863_Leaf | 1 | PgPLD-alpha1-7 |
| 863_Grains, 863_Immature Embryo, 863_Pericarp | 1 | PgPLD-p1-6 |
| 863_Endosperm, 863_Glumes, 863_Grains | 1 | PgPLA1-II-6c |
| 863_Endosperm, 863_Grains, 863_Pericarp | 4 | PgPLA2-3a, PgPLD-beta1-1b, PgPLD-alpha2-1, PgPLC-delta-4c |
| 863_Endosperm, 863_Immature Embryo, 863_Pericarp | 1 | PgPLC-delta-5 |
| 863_Grains, 863_Immature Embryo | 1 | PgPLD-delta1-2b |
| 863_Endosperm, 863_Grains | 1 | PgPLC-delta-1 |
| 863_Grains, 863_Pericarp | 2 | PgPLA1-DAD1-1a, PgPLA1-II-1b |
| 863_Immature Embryo, 863_Pericarp | 2 | PgPLA1-II-6b, PgPLA1-II-6a |
| 863_Endosperm, 863_Glumes | 1 | PgPLD-alpha2-5 |
| 863_Glumes, 863_Leaf | 1 | PgPLA1-II-6e |
| 863_Grains | 9 | PgPLA1-II-1a, PgPLD-alpha1-5, PgPLD-delta1-7a, PgPLD-alpha1-1, PLA1-beta2-4, PgPLA1-DAD1-1b, PgPLD-beta1-3b, PgPLD-delta1-2a, PgPLA1-gamma1 |
| 863_Glumes | 2 | PLA1-gamma-3, PgPLD-beta1-4a |

**3b. Details of phospholipases with an overlap in expression in different tissues of ICMB_95222 genotype**

| **Tissue type** | **No. of candidates expressed** | **List of candidates** |
| --- | --- | --- |
| 95222_Endosperm 95222_Glumes 95222_Grains 95222_Immature Embryo 95222_Leaf 95222_Pericarp | 5 | PgPLD-beta1-5b, PgPLD-p1-5, PgPLD-zeta5, PgPLA2-3b, PgPLC-delta-4b |
| 95222_Glumes 95222_Grains 95222_Immature Embryo 95222_Leaf 95222_Pericarp | 1 | PgPLD-delta1-7b |
| 95222_Endosperm 95222_Grains 95222_Immature Embryo 95222_Leaf 95222_Pericarp | 1 | PgPLD-alpha1-7 |
| 95222_Endosperm 95222_Grains 95222_Immature Embryo 95222_Pericarp | 1 | PgPLC-delta-4a |
| 95222_Endosperm 95222_Grains 95222_Immature Embryo 95222_Leaf | 1 | PgPLA1-II-6b |
| 95222_Glumes 95222_Grains 95222_Leaf 95222_Pericarp | 3 | PgPLD-p1-6, PgPLD-p1-1, PgPLD-alpha2-1 |
| 95222_Endosperm 95222_Grains 95222_Leaf 95222_Pericarp | 1 | PgPLC-delta-1 |
| 95222_Endosperm 95222_Glumes 95222_Leaf 95222_Pericarp | 1 | PgPLC-delta-5 |
| 95222_Grains 95222_Immature Embryo 95222_Leaf | 1 | PLA1-beta2-3 |
| 95222_Glumes 95222_Grains 95222_Leaf | 1 | PgPLA1-II-6c |
| 95222_Endosperm 95222_Grains 95222_Leaf | 1 | PgPLA1-II-1b |
| 95222_Grains 95222_Leaf 95222_Pericarp | 3 | PgPLA2-3a, PgPLA1-gamma1, PgPLC-delta-4c |
| 95222_Glumes 95222_Leaf 95222_Pericarp | 1 | PgPLA1-II-6d |
| 95222_Grains 95222_Leaf | 2 | PgPLA1-DAD1-1a, PgPLD-beta1-3b |
| 95222_Endosperm 95222_Immature Embryo | 1 | PgPLA1-II-6a |
| 95222_Glumes 95222_Leaf | 1 | PgPLA1-II-6e |
| 95222_Endosperm 95222_Pericarp | 1 | PgPLD-beta1-5a |
| 95222_Leaf 95222_Pericarp | 1 | PgPLD-beta1-1b |
| 95222_Grains | 10 | PgPLA1-II-1a, PgPLD-alpha1-5, PgPLD-delta1-7a, PgPLD-alpha1-1, PLA1-beta2-4, PgPLD-beta1-3a, PgPLA1-DAD1-1b, PgPLD-delta1-2a, PgPLD-beta1-7, PgPLD-beta1-1a |
| 95222_Immature Embryo | 1 | PgPLA2-2 |
| 95222_Glumes | 1 | PgPLD-alpha2-5 |
| 95222_Leaf | 4 | PgPLA2-1, PLA1-beta2-5, PgPLD-beta1-4a, PgPLD-delta1-2b |

**3c. List of phospholipases with specific/overlap in expression in grains of ICMB_863 and ICMB_95222 genotypes**

| **Tissue type** | **Total no. of phospholipases expressed** | **List of phospholipases expressed** |
| --- | --- | --- |
| 863_Grains 95222_Grains | 30 | PgPLD-delta1-7b PgPLA1-II-1a PgPLD-alpha1-5 PgPLD-beta1-5b PgPLD-delta1-7a PLA1-beta2-3 PgPLA1-DAD1-1a PgPLA2-3a PgPLD-p1-5 PgPLD-alpha1-1 PLA1-beta2-4 PgPLD-p1-6 PgPLD-zeta5 PgPLA2-3b PgPLD-beta1-3a PgPLA1-DAD1-1b PgPLD-alpha1-7 PgPLD-beta1-3b PgPLD-p1-1 PgPLD-delta1-2a PgPLD-alpha2-1 PgPLD-beta1-7 PgPLA1-gamma1 PgPLC-delta-4c PgPLD-beta1-1a PgPLA1-II-6c PgPLA1-II-1b PgPLC-delta-1 PgPLC-delta-4b |
| 863_Grains | 5 | PgPLD-alpha1-1, PgPLD-alpha1-5, PgPLD-delta1-7a, PgPLA1-II-1a, and PgPLD-delta1-2a |
| 95222_Grains | 5 | PgPLD-alpha1-1, PgPLD-alpha1-5, PgPLD-delta1-7a, PgPLA1-II-1a, and PgPLD-delta1-2a |

**3d. List of phospholipases with specific/overlap in expression in immature embryos of ICMB_863 and ICMB_95222 genotypes**

| **Tissue type** | **Total no. of phospholipases expressed** | **List of phospholipases expressed** |
| --- | --- | --- |
| 863_Immature Embryos 95222_Immature Embryos | 13 | PgPLD-delta1-7b PgPLD-alpha1-7 PgPLA2-2 PgPLD-beta1-5b PLA1-beta2-3 PgPLD-p1-5 PgPLC-delta-4a PgPLA1-II-6a PgPLD-zeta5 PgPLA2-3b PgPLA1-II-6b PgPLC-delta-4b |
| 863_Immature Embryos | 9 | PgPLD-beta1-5a PgPLC-delta-5a PgPLD-p1-1 PgPLD-p1-6 PgPLD-beta1-7 PgPLD-beta1-1a PgPLA1-II-6d PgPLD-beta1-3a PgPLD-delta1-2b |

**3e. List of phospholipases with specific/overlap in expression in glumes of ICMB_863 and ICMB_95222 genotypes**

| **Tissue type** | **Total no. of phospholipases expressed** | **List of phospholipases expressed** |
| --- | --- | --- |
| 863_Glumes 95222_Glumes | 10 | PgPLD-delta1-7b PgPLD-p1-5 PgPLD-alpha2-5 PgPLD-zeta5 PgPLA1-II-6c PgPLA2-3b PgPLA1-II-6d PgPLA1-II-6e PgPLC-delta-4b |
| 863_Glumes | 5 | PgPLD-beta1-5a PLA1-beta2-3 PgPLC-delta-4a PgPLD-beta1-4a PLA1-gamma-3 |
| 95222_Glumes | 5 | PgPLD-beta1-5b PgPLC-delta-5a PgPLD-p1-1 PgPLD-alpha2-1 PgPLD-p1-6 |

**3f. List of phospholipases with specific/overlap in expression in endosperm of ICMB_863 and ICMB_95222 genotypes**

| **Tissue type** | **Total no. of phospholipases expressed** | **List of phospholipases expressed** |
| --- | --- | --- |
| 863_Endosperm 95222_Endosperm | 11 | PgPLD-beta1-5a PgPLD-alpha1-7 PgPLD-beta1-5b PgPLC-delta-5a PgPLD-p1-5 PgPLC-delta-4a PgPLD-zeta5 PgPLA2-3b PgPLC-delta-1 PgPLC-delta-4b |
| 863_Endosperm | 13 | PgPLD-delta1-7b PgPLA2-2 PgPLA2-3a PgPLD-alpha2-5 PgPLD-p1-1 PgPLD-alpha2-1 PgPLD-beta1-7 PgPLC-delta-4c PgPLD-beta1-1a PgPLA1-II-6c PgPLA1-II-6d PgPLD-beta1-3a PgPLD-beta1-1b |
| 95222_Endosperm | 3 | PgPLA1-II-6a PgPLA1-II-6b PgPLA1-II-1b |

**3g. List of phospholipases with specific/overlap in expression in the pericarp of ICMB_863 and ICMB_95222 genotypes**

| **Tissue type** | **Total no. of phospholipases expressed** | **List of phospholipases expressed** |
| --- | --- | --- |
| 863_Pericarp 95222_Pericarp | 17 | PgPLD-delta1-7b PgPLD-beta1-5a PgPLD-beta1-5b PgPLC-delta5 PgPLA2-3a PgPLD-p1-5 PgPLC-delta-4a PgPLD-p1-1 PgPLD-alpha2-1 PgPLD-p1-6 PgPLD-zeta5 PgPLC-delta-4c PgPLA2-3b PgPLA1-II-6d PgPLC-delta-4b PgPLD-beta1-1b |
| 863_Pericarp | 9 | PgPLA2-2 PLA1-beta2-3 PgPLA1-DAD1-1a PgPLA1-II-6a PgPLD-beta1-7 PgPLD-beta1-1a PgPLD-beta1-3a PgPLA1-II-6b PgPLA1-II-1b |
| 95222_Pericarp | 3 | PgPLD-alpha1-7 PgPLA1-gamma1 PgPLC-delta-1 |

**3h. List of phospholipases with specific/overlap in expression in the leaf of ICMB_863 and ICMB_95222 genotypes**

| **Tissue type** | **Total no. of phospholipases expressed** | **List of phospholipases expressed** |
| --- | --- | --- |
| 863_Leaf 95222_Leaf | 7 | PgPLD-alpha1-7 PLA1-beta2-3 PgPLD-zeta5 PgPLA2-3b PgPLA1-II-6e PgPLC-delta-4b |
| 863_Leaf | 1 | PgPLC-delta-4a |
| 95222_Leaf | 22 | PgPLD-delta1-7b PgPLD-beta1-5b PgPLC-delta-5a PgPLA1-DAD1-1a PgPLA2-3a PgPLD-p1-5 PgPLD-beta1-3b PgPLD-beta1-4a PgPLD-p1-1 PgPLD-alpha2-1 PgPLD-p1-6 PgPLA1-gamma1 PgPLC-delta-4c PgPLA1-II-6c PgPLA1-II-6d PgPLD-delta1-2b PgPLA1-II-6b PgPLA1-II-1b PgPLA2-1 PLA1-beta2-5 PgPLC-delta-1 PgPLD-beta1-1b |
